# Supplementary material for: Quantum majorization and a complete set of entropic conditions for quantum thermodynamics
Source: Nat Commun. 2018 Dec 17;9:5352. doi: 10.1038/s41467-018-06261-7 (PMC6297236; doi:10.1038/s41467-018-06261-7)
Supplement: Supplementary file 1 — Supplementary Information [file 41467_2018_6261_MOESM1_ESM.pdf]

Supplementary Material
# – Quantum majorization and a complete set of entropic conditions for quantum thermodynamics

Gilad Gour, David Jennings, Francesco Buscemi, Runyao Duan, and Iman Marvian

SUPPLEMENTARY NOTE 1: PROOF OF THEOREM 1

For completeness we repeat the statement of theorem 1.
Theorem 1 Let $\rho^{\mathrm{AB}} \in \mathcal{B}(\mathcal{H}_{\mathrm{A}} \otimes \mathcal{H}_{\mathrm{B}})$ and $\sigma^{\mathrm{AC}} \in \mathcal{B}(\mathcal{H}_{\mathrm{A}} \otimes \mathcal{H}_{\mathrm{C}})$ be two compatible bipartite quantum states. Let $\{M_j^{\mathrm{A}}\}$ be an arbitrary, but fixed, informationally complete POVM on system A. Denote the dimension of any system X as $d_X \in \mathbb{N}$. The following are equivalent:

1. The state ρ^{AB} quantum majorizes σ^{AC},
$$\sigma^{\mathrm{AC}} \prec_q \rho^{\mathrm{AB}}. \quad (1)$$
2. For any quantum process (CPTP linear map) $\Phi : \mathcal{B}(\mathcal{H}_{\mathrm{A}}) \to \mathcal{B}(\mathcal{H}_{\mathrm{A}'})$, with $d_{\mathrm{A}'} = d_{\mathrm{C}}$,
$$H_{\min}(A'|B)_{\Phi\otimes\mathsf{id}(\rho^{\mathrm{AB}})} \leqslant H_{\min}(A'|C)_{\Phi\otimes\mathsf{id}(\sigma^{\mathrm{AC}})} \quad (2)$$
3. Supplementary Equation (2) holds for any measure-and-prepare quantum channel $\Phi : \mathcal{B}(\mathcal{H}_{\mathrm{A}}) \to \mathcal{B}(\mathcal{H}_{\mathrm{A}'})$ of the form:
$$\Phi\left(\eta^{\mathrm{A}}\right) = \sum_{j=1}^{d_{\mathrm{A}}^2} \mathrm{Tr}\left[M_j^{\mathrm{A}} \eta^{\mathrm{A}}\right] \omega_j^{\mathrm{A}'}\ , \quad (3)$$
while the states $\{\omega_j^{\mathrm{A}'}\}$ can freely vary.
4. $g(\rho^{\mathrm{AB}}, \sigma^{\mathrm{AC}}) \geqslant 1$, where the the function g is defined by the following semidefinite programming:

$$g(\rho^{\mathrm{AB}}, \sigma^{\mathrm{AC}}) = \max \left\{ y \;\middle|\; \forall j \;\; y\sigma_j^{\mathrm{T}} \leqslant \mathrm{Tr}_{\mathrm{B}}\left[\tau^{CB}(I \otimes \rho_j)\right],\ \tau^{CB} \geqslant 0,\ \tau^{\mathrm{B}} \leqslant I \right\} \quad (4)$$

where
$$\rho_j \equiv \frac{\mathrm{Tr}_{\mathrm{A}}\left[\left(M_j^{\mathrm{A}} \otimes \mathbb{1}^{\mathrm{B}}\right)\rho^{\mathrm{AB}}\right]}{\mathrm{Tr}\left[M_j^{\mathrm{A}} \rho^{\mathrm{A}}\right]} \text{ and}$$
$$\sigma_j \equiv \frac{\mathrm{Tr}_{\mathrm{A}}\left[\left(M_j^{\mathrm{A}} \otimes \mathbb{1}^{\mathrm{C}}\right)\sigma^{\mathrm{AC}}\right]}{\mathrm{Tr}\left[M_j^{\mathrm{A}} \sigma^{\mathrm{A}}\right]}. \quad (5)$$

In order to prove Theorem 1, we will begin by proving the following lemma. Recall the definition of conditional min-entropy $H_{\min}(A|B)_\Omega$, of a bipartite state Ω^{AB},
$$H_{\min}(A|B)_\Omega := -\log \inf_{\tau^{\mathrm{B}} \geqslant 0} \{\mathrm{Tr}\left[\tau^{\mathrm{B}}\right] : \mathbb{1}^{\mathrm{A}} \otimes \tau^{\mathrm{B}} \geqslant \Omega^{\mathrm{AB}}\}. \quad (6)$$

Lemma 1 Let $\{\rho_i^{\mathrm{B}}\}_{i=1}^n$ and $\{\sigma_i^{\mathrm{C}}\}_{i=1}^n$ be two sets of n density matrices in $\mathcal{B}(\mathcal{H}_{\mathrm{B}})$ and $\mathcal{B}(\mathcal{H}_{\mathrm{C}})$, respectively. Let $\{q_i\}_{i=1}^n$ be some arbitrary but fixed probability distribution with $q_i > 0$. For any set of n density matrices $\{\omega_i^{\mathrm{A}}\}_{i=1}^n$ in $\mathcal{B}(\mathcal{H}_{\mathrm{A}})$ (with $d_{\mathrm{A}} = d_{\mathrm{C}}$) define the following tripartite separable matrix:
$$\Omega^{ABC} \equiv \sum_{i=1}^n q_i\, \omega_i^{\mathrm{A}} \otimes \rho_i^{\mathrm{B}} \otimes \sigma_i^{\mathrm{C}}\ . \quad (7)$$

Then, the following are equivalent:

1. There exists a CPTP map $\mathcal{E} : \mathcal{B}(\mathcal{H}_{\mathrm{B}}) \to \mathcal{B}(\mathcal{H}_{\mathrm{C}})$ such that
$$\mathcal{E}(\rho_i^{\mathrm{B}}) = \sigma_i^{\mathrm{C}} \quad \forall\, i = 1, ..., n\ . \quad (8)$$
2. For any $\omega_1^{\mathrm{A}}, ..., \omega_n^{\mathrm{A}} \in \mathcal{B}(\mathcal{H}_{\mathrm{A}})$:
$$2^{-H_{\min}(A|B)_\Omega} \geqslant d_{\mathrm{C}} \langle \phi_+^{\mathrm{AC}} | \Omega^{\mathrm{AC}} | \phi_+^{\mathrm{AC}} \rangle\ , \quad (9)$$
where $|\phi_+^{\mathrm{AC}}\rangle$ denotes the maximally entangled state on $\mathcal{H}_{\mathrm{A}} \otimes \mathcal{H}_{\mathrm{C}}$.
3. For any $\omega_1^{\mathrm{A}}, ..., \omega_n^{\mathrm{A}} \in \mathcal{B}(\mathcal{H}_{\mathrm{A}})$:
$$H_{\min}(A|B)_\Omega \leqslant H_{\min}(A|C)_\Omega\ . \quad (10)$$

Proof. Consider two families of density matrices, $\{\rho_i^{\mathrm{B}}\}_{i=1}^n$ and $\{\sigma_i^{\mathrm{C}}\}_{i=1}^n$. We want to reformulate, in an equivalent way, the condition
$$\exists \text{ CPTP } \mathcal{E} : \mathcal{E}(\rho_i^{\mathrm{B}}) = \sigma_i^{\mathrm{C}}, \forall i. \quad (11)$$
By introducing a set of self-adjoint operators $\{X_j^{\mathrm{C}}\}$ forming a basis for $\mathcal{B}(\mathcal{H}_{\mathrm{C}})$, Supplementary Equation (8) can be written as
$$\exists \text{ CPTP } \mathcal{E} : \mathrm{Tr}\left[\mathcal{E}(\rho_i^{\mathrm{B}})\ X_j^{\mathrm{C}}\right] = \mathrm{Tr}\left[\sigma_i^{\mathrm{C}}\ X_j^{\mathrm{C}}\right], \forall i, j. \quad (12)$$

Let us now consider the set of real vectors
$$\boldsymbol{r}_{\mathcal{E}} = (r_{ij}) : r_{ij} = \mathrm{Tr}\left[\mathcal{E}(\rho_i^{\mathrm{B}})\ X_j^{\mathrm{C}}\right] \quad (13)$$
obtained by letting $\mathcal{E}$ vary over all possible CPTP maps from system B to system C, while the ρ_i's and the X_j's are kept fixed. It is clear that the set
$$\mathcal{S} = \{\boldsymbol{r}_{\mathcal{E}} : \mathcal{E} \text{ CPTP}\} \quad (14)$$
is a closed and bounded convex set, as it is the image, under a linear map, of the set of CPTP maps from B to

C (that is a closed and bounded convex set). By writing $\boldsymbol{s} = (s_{ij})$ when $s_{ij} = \mathrm{Tr}\big[\sigma_i^{\mathrm{C}}\ X_j^{\mathrm{C}}\big]$, Supplementary Equation (12) becomes

$$\boldsymbol{s} \in \mathcal{S}\ . \tag{15}$$

At this point, we invoke the separation theorem for convex sets (see, e.g., Ref. [1]), which in particular implies the following:

Lemma [Separation theorem] Let $\mathcal{S} \subset \mathbb{R}^n$ be a closed and bounded convex set. The vector $y \in \mathbb{R}^n$ belongs to $\mathcal{S}$, i.e. $y \in \mathcal{S}$, if and only if, for any vector $k \in \mathbb{R}^n$, $\max_{x\in\mathcal{S}} k \cdot x \geqslant k \cdot y$.

Applied to our case, it yields that condition (8) is equivalent to

$$\forall \boldsymbol{\lambda} = (\lambda_{ij}) \text{ with } \lambda_{ij} \in \mathbb{R}, \quad \max_{\boldsymbol{r}\in\mathcal{S}} \boldsymbol{r} \cdot \boldsymbol{\lambda} \geqslant \boldsymbol{s} \cdot \boldsymbol{\lambda}\ , \tag{16}$$

namely,

$$\begin{aligned}&\forall \boldsymbol{\lambda} = (\lambda_{ij}) \text{ with } \lambda_{ij} \in \mathbb{R},\\ &\max_{\mathcal{E}:\mathrm{CPTP}} \sum_{ij} \lambda_{ij}\, \mathrm{Tr}\big[\mathcal{E}(\rho_i^{\mathrm{B}})\ X_j^{\mathrm{C}}\big] \geqslant \sum_{ij} \lambda_{ij}\, \mathrm{Tr}\big[\sigma_i^{\mathrm{C}}\ X_j^{\mathrm{C}}\big]\ .\end{aligned} \tag{17}$$

Defining self-adjoint operators $Z_i^{\mathrm{C}} = \sum_j \lambda_{ij} X_j^{\mathrm{C}}$, we can reformulate the statement as follows:

$$\begin{aligned}&\forall \text{ self-adjoint } \{Z_i^{\mathrm{C}}\},\\ &\max_{\mathcal{E}:\mathrm{CPTP}} \sum_i \mathrm{Tr}\big[\mathcal{E}(\rho_i^{\mathrm{B}})\ Z_i^{\mathrm{C}}\big] \geqslant \sum_i \mathrm{Tr}\big[\sigma_i^{\mathrm{C}}\ Z_i^{\mathrm{C}}\big]\ .\end{aligned} \tag{18}$$

In this condition we should vary operators Z_i^{C} over all self-adjoint operators. However, it turns out that we can only restrict these operators to the set of density operators. In other words, this condition is equivalent to

$$\begin{aligned}&\forall \text{ states } \{\omega_i^{\mathrm{C}}\},\\ &\max_{\mathcal{E}:\mathrm{CPTP}} \sum_i \mathrm{Tr}\big[\mathcal{E}(\rho_i^{\mathrm{B}})\ \omega_i^{\mathrm{C}}\big] \geqslant \sum_i \mathrm{Tr}\big[\sigma_i^{\mathrm{C}}\ \omega_i^{\mathrm{C}}\big]\ .\end{aligned} \tag{19}$$

To show this note that for any bounded self-adjoint operator Z_i^{C} and positive number $z_i > \|Z_i^{\mathrm{C}}\|_\infty$, $Z_i^{\mathrm{C}} + z_i \mathbb{1}^{\mathrm{C}}$ is a positive operator, and therefore $\omega_i^{\mathrm{C}} \equiv (Z_i^{\mathrm{C}} + z_i\mathbb{1}^{\mathrm{C}})/\,\mathrm{Tr}\big[Z_i^{\mathrm{C}} + z_i\mathbb{1}^{\mathrm{C}}\big]$ is a density operator. Furthermore, since constants z_i can be chosen independent of each other, we can choose them such that $\mathrm{Tr}\big[Z_i^{\mathrm{C}} + z_i\mathbb{1}^{\mathrm{C}}\big]$ is a positive constant independent of i. Then, putting this set of states ω_i^{C} into Supplementary Eq. (19) and using the fact that $\mathrm{Tr}\big[\mathcal{E}(\rho_i^{\mathrm{B}})\big] = \mathrm{Tr}\big[\sigma_i^{\mathrm{C}}\big] = 1$ we can recover Supplementary Eq. (18).

In Supplementary Eq.(19) all the terms corresponding to different states ω_i have equal weights in the summations. Next, we show how this constraint can be relaxed: Consider an arbitrary fixed probability distribution q_i, with full support, such that $q_{\min} \equiv \min_i q_i > 0$. Then, it turns out that condition (19) can be reformulated as:

$$\begin{aligned}&\forall \text{ states } \{\omega_i^{\mathrm{C}}\},\\ &\max_{\mathcal{E}:\mathrm{CPTP}} \sum_i q_i\, \mathrm{Tr}\big[\mathcal{E}(\rho_i^{\mathrm{B}})\ \omega_i^{\mathrm{C}}\big] \geqslant \sum_i q_i\, \mathrm{Tr}\big[\sigma_i^{\mathrm{C}}\ \omega_i^{\mathrm{C}}\big]\ .\end{aligned} \tag{20}$$

To show this we use the fact that for any state ω_i^{C}, the convex combination $(q_{\min}/q_i)\omega_i^{\mathrm{C}} + (1 - q_{\min}/q_i)\mathbb{1}^{\mathrm{C}}/d_{\mathrm{C}}$ is also a valid state. Using this together with the fact that $\mathrm{Tr}\big[\mathcal{E}(\rho_i^{\mathrm{B}})\big] = \mathrm{Tr}\big[\sigma_i^{\mathrm{C}}\big] = 1$ we can derive condition (19) from condition (20) and vice versa.

The next step is to introduce an auxiliary system $A \cong C$ (i.e., $d_{\mathrm{A}} = d_{\mathrm{C}}$), choose two orthonormal bases $\{|i_{\mathrm{A}}\rangle\}$ and $\{|i_{\mathrm{C}}\rangle\}$, and define the maximally entangled state

$$|\phi_+^{\mathrm{AC}}\rangle \equiv d_{\mathrm{A}}^{-1/2} \sum_{i=1}^{d_{\mathrm{A}}} |i_{\mathrm{A}}\rangle|i_{\mathrm{C}}\rangle\ . \tag{21}$$

Noticing that $\mathrm{Tr}[XY] = d\,\mathrm{Tr}\big[X \otimes Y^{\mathrm{T}}\ \phi_+\big]$, where the superscript T denotes the transposition with respect to the basis in (21), and that ω_i are density matrices if and only if $(\omega_i)^{\mathrm{T}}$ are, we arrive at

$$\begin{aligned}&\forall \text{ states } \{\omega_i^{\mathrm{A}}\},\\ &\max_{\mathcal{E}:\mathrm{CPTP}} \sum_i q_i\, \mathrm{Tr}\big[\big\{\omega_i^{\mathrm{A}} \otimes \mathcal{E}(\rho_i^{\mathrm{B}})\big\}\ \phi_+^{\mathrm{AC}}\big]\\ &\geqslant \sum_i q_i\, \mathrm{Tr}\big[\big\{\omega_i^{\mathrm{A}} \otimes \sigma_i^{\mathrm{C}}\big\}\ \phi_+^{\mathrm{AC}}\big]\ .\end{aligned} \tag{22}$$

As shown in Ref. [5], the quantity

$$\max_{\mathcal{E}:\mathrm{CPTP}} \sum_i q_i\, \mathrm{Tr}\big[\big\{\omega_i^{\mathrm{A}} \otimes \mathcal{E}(\rho_i^{\mathrm{B}})\big\}\ \phi_+^{\mathrm{AC}}\big] \tag{23}$$

$$= \max_{\mathcal{E}:\mathrm{CPTP}} \langle\phi_+^{\mathrm{AC}}|(\mathsf{id} \otimes \mathcal{E})(\Omega^{\mathrm{AB}})|\phi_+^{\mathrm{AC}}\rangle\ , \tag{24}$$

for $\Omega^{\mathrm{AB}} \equiv \sum_i q_i\ \omega_i^{\mathrm{A}} \otimes \rho_i^{\mathrm{B}}$, can be written in terms of the conditional min-entropy (6) as

$$\frac{1}{d_{\mathrm{A}}} 2^{-H_{\min}(A|B)_\Omega}\ . \tag{25}$$

We thus proved that statements (1) and (2) of Lemma 1 are indeed equivalent.

Moreover, a *sufficient* condition for (8) is that

$$\begin{aligned}&\forall \text{ states } \{\omega_i^{\mathrm{A}}\},\\ &\max_{\mathcal{E}:\mathrm{CPTP}} \sum_i q_i\, \mathrm{Tr}\big[\big\{\omega_i^{\mathrm{A}} \otimes \mathcal{E}(\rho_i^{\mathrm{B}})\big\}\ \phi_+^{\mathrm{AC}}\big]\\ &\geqslant \max_{\mathcal{F}:\mathrm{CPTP}} \sum_i q_i\, \mathrm{Tr}\big[\big\{\omega_i^{\mathrm{A}} \otimes \mathcal{F}(\sigma_i^{\mathrm{C}})\big\}\ \phi_+^{\mathrm{AC}}\big]\ ,\end{aligned} \tag{26}$$

namely

$$2^{-H_{\min}(A|B)_\Omega} \geqslant 2^{-H_{\min}(A|C)_\Omega}\ , \tag{27}$$

where now $\Omega^{\rm AB}$ and $\Omega^{\rm AC}$ are meant as the marginals of the same tripartite extension $\Omega^{ABC} = \sum_i q_i\ \omega_i^{\rm A} \otimes \rho_i^{\rm B} \otimes \sigma_i^{\rm C}$. However, it is easy to verify that the above condition is also necessary: indeed, if (8) holds, due to the data-processing theorem applied to the conditional min-entropy (see, e.g., Ref. [6] and [7]), $H_{\min}(A|B)_\Omega \leqslant H_{\min}(A|C)_\Omega$. We thus have that statements (1) and (3) are also logically equivalent, and hence the proof is complete. ∎

We are now ready to prove the main theorem.

Proof of Theorem 1:

Let $\{Q_k^{\rm A}\}_{k=1}^{d_{\rm A}^2}$ be the dual basis of $\{M_j^{\rm A}\}$ in $\mathcal{B}(\mathcal{H}_{\rm A})$, that is, $\mathrm{Tr}\left[M_j^{\rm A} Q_k^{\rm A}\right] = \delta_{jk}$. Then, since $\{Q_k^{\rm A}\}$ is itself a basis, we can write

$$\rho^{\rm AB} = \sum_{k=1}^{d_{\rm A}^2} Q_k^{\rm A} \otimes \widetilde{\rho}_k^{\rm B} \quad \text{and} \quad \sigma^{\rm AC} = \sum_{k=1}^{d_{\rm A}^2} Q_k^{\rm A} \otimes \widetilde{\sigma}_k^{\rm C} \tag{28}$$

where

$$\begin{aligned} \widetilde{\rho}_j^{\rm B} &\equiv \mathrm{Tr}_{\rm A}\left[\left(M_j^{\rm A} \otimes \mathbb{1}^{\rm B}\right)\rho^{\rm AB}\right] \\ \widetilde{\sigma}_j^{\rm C} &\equiv \mathrm{Tr}_{\rm A}\left[\left(M_j^{\rm A} \otimes \mathbb{1}^{\rm C}\right)\sigma^{\rm AC}\right] \end{aligned} \tag{29}$$

are sub-normalized quantum states (i.e. positive semidefinite matrices). Moreover, since $\rho^{\rm A} = \sigma^{\rm A}$ we have $\mathrm{Tr}\left[\widetilde{\rho}_j^{\rm B}\right] = \mathrm{Tr}\left[\widetilde{\sigma}_j^{\rm C}\right] \equiv p_j$. We therefore conclude that that there exists CPTP map $\mathcal{E}$ that satisfies Supplementary Equation (1) if and only if there exists a CPTP map $\mathcal{E}$ that satisfies

$$\sigma_j^{\rm C} = \mathcal{E}\left(\rho_j^{\rm B}\right) \tag{30}$$

where $\rho_j^{\rm B} \equiv \widetilde{\rho}_j^{\rm B}/p_j$ and $\sigma_j^{\rm C} \equiv \widetilde{\sigma}_j^{\rm C}/p_j$. To apply Lemma 1, we introduce a system A' with $d_{\rm A'} = d_{\rm C}$, we fix an arbitrary probability distribution $q_i > 0$, and define

$$\begin{aligned} \Omega^{A'BC} \equiv \sum_{j=1}^{d_{\rm A}^2} q_j\ \omega_j^{\rm A'} \otimes \rho_j^{\rm B} \otimes \sigma_j^{\rm C} = \sum_{j=1}^{d_{\rm A}^2} \frac{q_j}{p_j^2} \times \\ \omega_j^{\rm A'} \otimes \mathrm{Tr}_{\rm A}\left[\left(M_j^{\rm A} \otimes I^{\rm B}\right)\rho^{\rm AB}\right] \otimes \mathrm{Tr}_{\rm A}\left[\left(M_j^{\rm A} \otimes I^{\rm C}\right)\sigma^{\rm AC}\right]\ , \end{aligned} \tag{31}$$

where the states $\omega_i^{\rm A'}$ can vary. Then, taking $q_j = p_j$, we conclude that

$$\begin{aligned} \Omega^{A'B} &= \Phi \otimes \mathsf{id}\left(\rho^{\rm AB}\right)\ , \\ \Omega^{A'C} &= \Phi \otimes \mathsf{id}\left(\sigma^{\rm AC}\right)\ . \end{aligned} \tag{32}$$

Notice that, in case some $p_i = 0$, we can redefine the measurement operators $M_j^{\rm A} \to M_j^{\rm A} + \delta \mathbb{1}^{\rm A}$, in such a way that they still span the set $\mathcal{B}(\mathcal{H}_{\rm A})$ but have non-zero probability everywhere. With Supplementary Eq. (32) at hand, the proof of Theorem 1 follows now from Lemma 1. This completes the proof of theorem 1.

SUPPLEMENTARY NOTE 2: COMPLEXITY OF DECIDING QUANTUM MAJORIZATION

We will show now that the problem of whether there exists a CPTP map $\mathcal{E}$ such that $\mathcal{E}(\rho_i) = \sigma_i$ (see Lemma 1 above) can be formulated as a semidefinite programming. Following similar lines, also all the other versions of quantum majorization discussed in this paper can be shown to be equivalent to a semidefinite programming.

We start by noting that Supplementary Equation (9) can be written as

$$2^{-H_{\min}(A|B)_\Omega} \geqslant \sum_{i=1}^n q_i \mathrm{Tr}\left(\omega_i \sigma_i^{\rm T}\right) \tag{33}$$

where

$$\Omega^{\rm AB} = \sum_{i=1}^n q_i\ \omega_i \otimes \rho_i\ . \tag{34}$$

In the following we absorb the q_is into ω_is, so that the ω_is become subnormalized, satisfying $\sum_{i=1}^n \mathrm{Tr}[\omega_i] = 1$. We get that the above condition is equivalent to the condition $\alpha(t) \geqslant 1$ for all t, where

$$\begin{aligned} \alpha(t) \equiv & \frac{1}{t} \min \mathrm{Tr}[\tau] \\ & \text{subject to} \quad I^{\rm A} \otimes \tau \geqslant \sum_{i=1}^n \omega_i \otimes \rho_i, \\ & \sum_{i=1}^n \mathrm{Tr}\left(\sigma_i^{\rm T} \omega_i\right) = t\ ;\ \sum_{i=1}^n \mathrm{Tr}[\omega_i] = 1\ , \end{aligned} \tag{35}$$

with $\omega_i \geqslant 0$. After rescaling $\tau' \equiv \frac{1}{t}\tau$ and $\omega_i' \equiv \frac{1}{t}\omega_i$ we get

$$\begin{aligned} \alpha(t) \equiv & \min \mathrm{Tr}[\tau'] \\ & \text{subject to} \quad I^{\rm A} \otimes \tau' \geqslant \sum_{i=1}^n \omega_i' \otimes \rho_i, \\ & \sum_{i=1}^n \mathrm{Tr}\left(\sigma_i^{\rm T} \omega_i'\right) = 1\ ;\ \sum_{i=1}^n \mathrm{Tr}[\omega_i'] = 1/t\ , \end{aligned} \tag{36}$$

The condition $\alpha(t) \geqslant 1$ for all t is therefore equivalent to one condition, $\alpha \geqslant 1$ (more precisely, $\alpha = 1$ since it can be shown that α can never exceed 1), where

$$\begin{aligned} \alpha \equiv & \min \mathrm{Tr}[Z] \\ & \text{subject to} \quad I^{\rm A} \otimes Z \geqslant \sum_{i=1}^n X_i \otimes \rho_i, \\ & \sum_{i=1}^n \mathrm{Tr}\left(\sigma_i^{\rm T} X_i\right) = 1\ ;\ X_i \geqslant 0\ . \end{aligned} \tag{37}$$

We now show that the above minimization problem is an SDP. To see it, we define the following vector space, which is a direct sum of $n+2$ Hilbert spaces:

$$V_1 \equiv \mathrm{B}(\mathcal{H}^{\mathrm{A}}\otimes\mathcal{H}^{\mathrm{B}})\oplus\mathrm{B}(\mathcal{H}^{\mathrm{B}})\oplus\mathrm{B}(\mathcal{H}^{\mathrm{A}})\oplus\cdots\oplus\mathrm{B}(\mathcal{H}^{\mathrm{A}})\ . \tag{38}$$

The vector space V_1 is consisting of matrices $\zeta \in V_1$ of the form:

$$\zeta = (\eta, Z, X_1, ..., X_n) \tag{39}$$

where $\eta \in \mathrm{B}(\mathcal{H}^{\mathrm{A}} \otimes \mathcal{H}^{\mathrm{B}})$, $Z \in \mathrm{B}(\mathcal{H}^{\mathrm{B}})$, and $X_i \in \mathrm{B}(\mathcal{H}^{\mathrm{A}})$ for each $i = 1, ..., n$. In addition, we define the vector space $V_2 \equiv \mathrm{B}(\mathcal{H}^{\mathrm{A}} \otimes \mathcal{H}^{\mathrm{B}})$, and a linear transformation $\Gamma : V_1 \to V_2$ given by:

$$\Gamma(\zeta) = I \otimes Z - \sum_{i=1}^{n} X_i \otimes \rho_i - \eta\ . \tag{40}$$

Clearly, the map above is linear. Set $\sigma \equiv (\mathbf{0}, \mathbf{0}, \sigma_1^{\mathrm{T}}, ..., \sigma_n^{\mathrm{T}})$ so that $\mathrm{Tr}[\sigma\zeta] = \sum_{i=1}^{n} \mathrm{Tr}\left(\sigma_i^{\mathrm{T}} X_i\right)$. We also denote $C \equiv (\mathbf{0}, I, \mathbf{0}, ..., \mathbf{0})$. With these notations:

$$\alpha = \min\left\{\mathrm{Tr}[C\zeta]\ \middle|\ \zeta \geqslant 0\ ;\ \Gamma(\zeta) = 0\ ,\ \mathrm{Tr}[\sigma\zeta] = 1\right\} \tag{41}$$

To bring the above optimization problem to a canonical SDP form, we denote $H_j \equiv \Gamma^*(E_j)$, where $j = 1, ..., d_{\mathrm{A}}^2 d_{\mathrm{B}}^2$ and E_j is a basis of $\mathrm{B}(\mathcal{H}^{\mathrm{A}}\otimes\mathcal{H}^{\mathrm{B}})$. We also denote $H_0 \equiv \sigma$. With this notations we get

$$\begin{aligned} \alpha \equiv &\min \mathrm{Tr}[C\zeta] \\ &\text{subject to} \quad \zeta \geqslant 0 \\ &\qquad\qquad \mathrm{Tr}\left(\zeta H_j\right) = \delta_{0,j} \quad j = 0, 1, ..., d_{\mathrm{A}}^2 d_{\mathrm{B}}^2 \end{aligned} \tag{42}$$

It is interesting to note that the dual problem is given by

$$\begin{aligned} \beta \equiv &\max y \\ &\text{subject to} \quad y\sigma + \Gamma^*(\tau^{\mathrm{AB}}) \leqslant C\ \ , \tau^{\mathrm{AB}} \in V_2. \end{aligned} \tag{43}$$

where the dual map Γ^* is given by:

$$\Gamma^*(\tau^{\mathrm{AB}}) = \left(-\tau^{\mathrm{AB}}, \tau^{\mathrm{B}}, -\mathrm{Tr}_{\mathrm{B}}\left[\tau^{\mathrm{AB}}(I\otimes\rho_1)\right], ..., -\mathrm{Tr}_{\mathrm{B}}\left[\tau^{\mathrm{AB}}(I\otimes\rho_n)\right]\right) \tag{44}$$

Therefore, the dual problem can be expressed as

$$\begin{aligned} \beta = &\max\ y \\ &\text{subject to} \quad \tau^{\mathrm{AB}} \geqslant 0\ ;\ \tau^{\mathrm{B}} \leqslant I\ ;\ \text{and}\ \forall\, i = 1, ..., n \\ &\qquad\qquad y\sigma_i^{\mathrm{T}} \leqslant \mathrm{Tr}_{\mathrm{B}}\left[\tau^{\mathrm{AB}}(I\otimes\rho_i)\right] \end{aligned} \tag{45}$$

Note that α (or β) can be commuted efficiently using standard SDP algorithms.

SUPPLEMENTARY NOTE 3: TWO FORMULATIONS: SEMIDEFINITE PROGRAMMING VERSUS A COMPLETE SET OF MONOTONES

As mentioned in Remark 3 of the main text, and as shown in Note 2 above, all the instances of quantum majorization considered in this paper can be formulated as semidefinite programs. These are well known for being efficiently solvable. One may be left wondering, then, about the role and relevance of the alternative formulation of quantum majorization that we provide in Theorem 1, in terms of an infinite set of inequalities between state monotones. Clearly, if the problem is just to decide whether quantum majorization holds or not, one should run the corresponding semi-definite program. However, the SDP formulation does not provide us any further insight about why a solution exists or not, nor does it tell us anything about the resources at stake and the way to quantify them. In other words, it does not tell us much about the physics behind quantum majorization.

Ideally, a resource theory should not only provide an efficient way to check whether a free transformation exists between two states, but also a way to measure resources as state functions. While the SDP formulation fulfills the former requirement, the formulation in terms of a complete set of monotones fulfills the latter.

The fact that here we find a complete set of monotones comprising infinitely many such functions is not an artifact of the present approach, but it is something that appears in many other contexts too. For example, already in classical statistics, the majorization relation with catalytic transformations (i.e., the "trumping" relation) is known to be equivalent to an inequality that must hold for all (uncountably many) Rényi entropies [30], and no discrete set of equivalent conditions is known. Again in the classical case, catalytic thermal operations have also been characterized in terms of an infinite set of "second laws" involving free energy functionals [2]. In the quantum theory of entanglement, when the local dimension is four or higher, it is known that an infinite number of entanglement monotones is not only sufficient but also necessary, in order to determine state conversion [3].

The characterization of quantum majorization in terms of a complete set of monotones, as we show in what follows, is also able to completely capture the notion of quantum thermal processes, with respect to both energy and coherence. This is non-trivial (and perhaps even surprising), given that it was shown that no direct analogue, in terms of "simple" free energy functionals, would ever be able to capture the subtle interplay between energy and coherence appearing in genuinely quantum thermal processes between non-commuting states [4]. It is hence a merit of our approach to circumvent this obstacle providing, at the same time, a novel insight into the theory. Indeed, the monotones constructed here are able to go be-

yond free energy functionals, by explicitly bringing into the picture an external reference system, with respect to which information about energy and time (i.e., coherence) is measured. Such an insight, that suggests also an intriguing physical picture behind quantum majorization, cannot be gained by looking at the SDP formulation alone.

Finally, the characterization in terms of monotones has, with respect to the SDP formulation, another advantage, which is due to the fact that our monotones can be expressed as min-conditional entropies [5, 6]. This allows us to apply, in principle, the powerful tools developed for single-shot quantum information theory [7] in order to study their behavior in the asymptotic scenario, something that we leave open for future investigations.

SUPPLEMENTARY NOTE 4: RE-DERIVATION OF THERMO-MAJORIZATION AS THE CLASSICAL CASE

Thermo-majorization generalizes ordinary majorization in a natural way [8–13]. Given two probability distributions $\mathbf{p} = (p_i)$ and $\mathbf{q} = (q_i)$ together with the Gibbs distribution $\boldsymbol{\gamma} = (\gamma_i) = (\frac{1}{Z}e^{-\beta E_i})$ at temperature $T = (k\beta)^{-1}$, we say that $\mathbf{p}$ thermo-majorizes $\mathbf{q}$ and write $\mathbf{p} \succ_T \mathbf{q}$ exactly when the following holds

$$\sum_k |p_k - t\gamma_k| \geqslant \sum_k |q_k - t\gamma_k|, \tag{46}$$

for all $t \geqslant 0$. This can be shown to be equivalent [8–11, 13, 22] to the existence of a stochastic map S such that $S\mathbf{p} = \mathbf{q}$ and $S\boldsymbol{\gamma} = \boldsymbol{\gamma}$. In what follows, we show that quantum majorization reduces to Thermo-majorization in the classical case. In particular, we will show that the conditions in Theorem 1 (specifically, Supplementary Equation (10) of Lemma 1) reduces to Supplementary Eq. (46). We first start with the semi-classical case.

The semi-classical case

In this case, we assume that the n states, $\{\sigma_i^{\rm C}\}$, in Lemma 1 commute with each other. Therefore, we can assume that they are all diagonal with respect to a fixed basis. We show now that this immediately implies that the n states $\{\omega_i\}$ in Lemma 1 can also be taken to be diagonal in the same basis. In fact, in the following lemma we show that if $\{\sigma_i^{\rm C}\}$ are all symmetric with respect to some group, then the states $\{\omega_i\}$ also have the same symmetry.

Lemma 2 Using the same notations as in Lemma 1, let $\Delta : {\rm B}(\mathcal{H}^{\rm C}) \to {\rm B}(\mathcal{H}^{\rm C})$ be a CPTP map, and suppose $\Delta(\sigma_i^{\rm T}) = \sigma_i^{\rm T}$ for all $i = 1, ..., n$. Then, in all the statements of Lemma 1 we can replace the set $\{\omega_i^{\rm A}\}$ with the set $\Delta^\dagger(\omega_i^{\rm A})$.

Remark 1. The lemma above is particularly interesting if the map Δ corresponds to some symmetry. That is, suppose the states $\{\sigma_i^{\rm T}\}$ satisfy $U_g\sigma_i^{\rm T}U_g^\dagger = \sigma_i^{\rm T}$ for any $g \in G$, where $\{U_g\}$ is some unitary representation of a compact group G. In this case, one can take Δ to be the G-twirling, and thereby assume that all the $\omega_i^{\rm A}$s of Lemma 1 are also symmetric with respect to the same representation of G.

Proof. The proof follows from the two sides of Supplementary Eq. (9). On one hand,

$$\begin{aligned} d_{\rm C}\langle\phi_+^{\rm AC}|\Omega^{\rm AC}|\phi_+^{\rm AC}\rangle &= \sum_i q_i \,{\rm Tr}\big[\sigma_i^{\rm T}\omega_i\big] \\ &= \sum_i q_i \,{\rm Tr}\big[\Delta(\sigma_i^{\rm T})\omega_i\big] \\ &= \sum_i q_i \,{\rm Tr}\big[\sigma_i^{\rm T}\Delta^\dagger(\omega_i)\big] \ , \end{aligned} \tag{47}$$

where $\Delta^\dagger$ is the dual (adjoint) unital map of Δ. On the other hand, if

$$I \otimes \tau \geqslant \sum_{i=1}^n q_i \,\omega_i^{\rm A} \otimes \rho_i^{\rm B} \tag{48}$$

for some non-normalized state τ, then since $\Delta^\dagger$ is a unital CP map we get

$$I \otimes \tau \geqslant \sum_{i=1}^n q_i \,\Delta^\dagger(\omega_i^{\rm A}) \otimes \rho_i^{\rm B} \ . \tag{49}$$

That is,

$$2^{-H_{\min}(A|B)_\Omega} \geqslant 2^{-H_{\min}(A|B)_{\Delta^\dagger\otimes{\rm id}(\Omega)}} \ . \tag{50}$$

Combining (47) and (50) with (9) we conclude that if (9) holds for all states of the form $\{\Delta^\dagger(\omega_i^{\rm A})\}$ then it holds for any set of n states $\{\omega_i^{\rm A}\}$. This completes the proof of lemma 2.

The case that we are interested here is the one in which all the σ_is are diagonal with respect to some fixed basis. This is the case considered in Corollary 1 of Ref. [21]. In this case, we can take Δ to be the completely decohering map with respect to the fix basis. Since the set$\{\Delta(\omega_i)\}$ consists of diagonal matrices, we can assume w.l.o.g. that all the ω_is in Lemma 1 are diagonal. We can therefore write

$$\omega_i^{\rm A} \equiv \sum_{x=1}^{d_{\rm A}} r_{x|i}|x\rangle\langle x| \tag{51}$$

so that

$$\Omega^{\rm AB} = \sum_{x=1}^{d_{\rm A}} |x\rangle\langle x| \otimes \sum_{i=1}^n q_i r_{x|i}\rho_i^{\rm B} \tag{52}$$

is a classical quantum state. It is well known that for classical quantum states, the conditional min-entropy can

be expressed in terms of a guessing probability [5]. In the case that $d_{\rm A}=2$ the conditional-min entropy of $\Omega^{\rm AB}$ can be further simplified and we get

$$\begin{aligned}&2^{-H_{\min}(A|B)_\Omega}\\&=\min_\tau\left\{{\rm Tr}[\tau]\;:\;\tau\geqslant\sum_{i=1}^n q_i r_{x|i}\rho_i^{\rm B}\quad\forall x=1,2\right\}\\&=\frac{1}{2}+\frac{1}{2}\left\|\sum_{i=1}^n q_i(r_{1|i}-r_{2|i})\rho_i^{\rm B}\right\|_1\ .\end{aligned}\tag{53}$$

However, even if the σ_is all commute, it is not enough in general to restrict the comparison only to two-dimensional auxiliary states ω_i, if the goal is that of showing the existence of a CPTP map achieving $\rho_i\to\sigma_i$. If such a restriction is made, what one can show is the existence of a weaker map, namely, a 2-statistical morphism [14, 21], but counterexamples have been shown for which neither a CPTP nor a PTP map exists [23].

There are two very important exceptions to this. The first is the case in which there are only two commuting states $\{\rho_1,\rho_2\}$ and two commuting states $\{\sigma_1,\sigma_2\}$, namely, the case of two classical dichotomies. In this case, already Blackwell showed that two-dimensional commuting states ω_i suffice [11].

The second exception is that of two pairs of qubit density matrices $\{\rho_1,\rho_2\}$ and $\{\sigma_1,\sigma_2\}$: even if these do not commute, again, two-dimensional commuting states ω_i suffice [22].

Thermo-majorization

In the completely classical case, in addition to the ω_is, also the set $\{\rho_i^{\rm B}\}$ consists of diagonal matrices. Denoting

$$\rho_i^{\rm B}\equiv\sum_{y=1}^{d_{\rm B}}s_{y|i}|y\rangle\langle y|\tag{54}$$

we get that

$$\Omega^{\rm AB}=\sum_{x=1}^{d_{\rm A}}p_{xy}|x\rangle\langle x|\otimes|y\rangle\langle y|\ \ ;\ \ p_{xy}\equiv\sum_{i=1}^n q_i r_{x|i}s_{y|i}\ .\tag{55}$$

Now, in this case, the conditional min-entropy is given by

$$\begin{aligned}&2^{-H_{\min}(A|B)_\Omega}\\&=\min_\tau\left\{{\rm Tr}[\tau]\;:\;I^{\rm A}\otimes\tau\geqslant\sum_{x,y}p_{xy}|x\rangle\langle x|\otimes|y\rangle\langle y|\right\}\\&=\sum_y\max_x p_{xy}=\sum_y\max_x\ \boldsymbol{r}_x\cdot\boldsymbol{s}_y\end{aligned}\tag{56}$$

where for each x and y, $\boldsymbol{r}_x$ is the n-dimensional vector whose components are $\{q_i r_{x|i}\}_{i=1}^n$, and $\boldsymbol{s}_y$ is the n-dimensional probability vector whose components are $\{s_{y|i}\}_{i=1}^n$. Similarly, denoting by

$$\sigma_i^{\rm C}\equiv\sum_{z=1}^{d_{\rm B}}t_{z|i}|z\rangle\langle z|\ ,\tag{57}$$

we conclude that

$$2^{-H_{\min}(A|C)_\Omega}=\sum_z\max_x\ \boldsymbol{r}_x\cdot\boldsymbol{t}_z\ ,\tag{58}$$

where $\boldsymbol{t}_z$ is the probability vector whose components are $t_{z|i}$. Therefore, in the classical case, the condition in (10) is equivalent to

$$\sum_y f(\boldsymbol{s}_y)\geqslant\sum_z f(\boldsymbol{t}_z)\tag{59}$$

for any sub-linear functional f of the form $f(\boldsymbol{s})=\max_x\ \boldsymbol{r}_x\cdot\boldsymbol{s}$. Note that $\sum_y\boldsymbol{s}_y=\sum_z\boldsymbol{t}_z=(1,1,...,1)^{\rm T}$.

Finally, to obtain themo-majorization, we consider the case $n=2$. That is, we have two input states ρ_1 and ρ_2, and two output states σ_1 and σ_2. We can think of ρ_2 and σ_2 as Gibbs states. Note that all the vectors $\boldsymbol{r}_x$, $\boldsymbol{s}_y$, and $\boldsymbol{t}_z$ are two-dimensional since $n=2$. Therefore, in this case, it is sufficient to consider in (59) only sub-linear functionals with two elements; that is, of the form $f(\boldsymbol{s})=\max\{\boldsymbol{r}_1\cdot\boldsymbol{s},\ \boldsymbol{r}_2\cdot\boldsymbol{s}\}$ (see [20] for more details). We therefore conclude that the condition in (10) is equivalent to

$$\sum_y\max\{\boldsymbol{r}_1\cdot\boldsymbol{s}_y,\ \boldsymbol{r}_2\cdot\boldsymbol{s}_y\}\geqslant\sum_z\max\{\boldsymbol{r}_1\cdot\boldsymbol{t}_z,\ \boldsymbol{r}_2\cdot\boldsymbol{t}_z\}\tag{60}$$

for all $\boldsymbol{r}_1,\boldsymbol{r}_2\in\mathbb{R}_+^2$. Using the relation $\max\{a,b\}=\frac{a+b}{2}+\frac{|a-b|}{2}$ for any two real numbers a and b, the equation above becomes equivalent to

$$\sum_y|(\boldsymbol{r}_1-\boldsymbol{r}_2)\cdot\boldsymbol{s}_y|\geqslant\sum_z|(\boldsymbol{r}_1-\boldsymbol{r}_2)\cdot\boldsymbol{t}_z|\tag{61}$$

where we used the fact that $\sum_y\boldsymbol{s}_z=\sum_z\boldsymbol{t}_z=(1,1,...,1)^{\rm T}$. Denoting by $\boldsymbol{r}_1-\boldsymbol{r}_2\equiv\begin{pmatrix}a\\b\end{pmatrix}\in\mathbb{R}^2$, the above equation becomes

$$\sum_y|as_{y|1}+bs_{y|2}|\geqslant\sum_z|at_{z|1}+bt_{z|2}|\tag{62}$$

Dividing by a and denoting $r\equiv -b/a$ we conclude that our condition in (10) reduces in the classical case to the thermo-majorization condition:

$$\sum_y|s_{y|1}-rs_{y|2}|\geqslant\sum_z|t_{z|1}-rt_{z|2}|\quad\forall r\geqslant 0\ ,\tag{63}$$

Note that there is an equality above if $r<0$ so we assume w.l.o.g. that $r\geqslant 0$.

Proof of Corollary 2

The proof of Corollary 2 can now be established. Suppose we are interested in the conversion of $\rho^{\rm A}$ into $\sigma^{\rm A'}$ under TPs. Moreover suppose that $[\rho^{\rm A}, \mathcal{H}^{\rm A}] = 0$, as explained in the main text one may restrict without loss of generality to η_1 and η_2 being incoherent in energy. Therefore the state $\Omega^{\rm RA}$ is a classical state. Since TPs are covariant, and $\rho^{\rm A}$ is incoherent in energy it implies that the states accessible under this class must also be incoherent in energy and so $[\sigma^{\rm A'}, H^{\rm A'}] = 0$ is a necessary condition. Since both input and output states are incoherent the problem reduces to the interconversion of the distributions over energy under stochastic maps that preserve the Gibbs state. This coincides with the conditions for thermo-majorization as stated above.

On the other hand, suppose $[\sigma^{\rm A'}, H^{\rm A'}] = 0$. Now if there exists a a TP map $\mathcal{E}$ such that $\mathcal{E}(\rho^{\rm A}) = \sigma^{\rm A'}$ it is readily seen that $U'(t)\mathcal{E}(\rho^{\rm A})U'(t)^\dagger = \mathcal{E}(U'(t)\rho^{\rm A}U'(t)^\dagger) = \sigma^{\rm A'}$ for any t. Averaging over t gives that $\mathcal{E}(\langle\rho^{\rm A}\rangle) = \sigma^{\rm A'}$. Therefore $\rho^{\rm A} \to \sigma^{\rm A'}$ under TPs if and only if $\langle\rho^{\rm A}\rangle \to \sigma^{\rm A'}$ under TPs. Therefore such an interconversion is possible if and only if the distribution over energy of $\langle\rho^{\rm A}\rangle$ thermo-majorizes the distribution over energy of $\sigma^{\rm A'}$.

SUPPLEMENTARY NOTE 5: G-COVARIANT MAPS

Theorem 1 can also be specialized to G-covariant maps. In what follows, we consider three unitary representations $g \to U_g$ of the same compact group G on systems A, B, and C. We use the following notations: $\mathcal{U}_g(x) = U_g x U_g^\dagger$, $\overline{\mathcal{U}}_g(x) = U_g^* x U_g^{\rm T}$, $\mathcal{U}_g^{\rm T}(x) = U_g^{\rm T} x U_g^*$, and $\mathcal{U}_g^\dagger(x) = U_g^\dagger x U_g$, with obvious meaning of symbols. We also introduce the bipartite twirling operation

$$\mathcal{G}(x) = \int_G dg\, \overline{\mathcal{U}}_g \otimes \mathcal{U}_g(x) \tag{64}$$

G-covariant version of Lemma 1

Lemma 3 Let $\{\rho_i^{\rm B}\}_{i=1}^n$ and $\{\sigma_i^{\rm C}\}_{i=1}^n$ be two sets of n density matrices in $\mathcal{B}(\mathcal{H}_{\rm B})$ and $\mathcal{B}(\mathcal{H}_{\rm C})$, respectively. Let $\{q_i\}_{i=1}^n$ be some arbitrary but fixed probability distribution with $q_i > 0$. For any set of n density matrices $\{\omega_i^{\rm A}\}_{i=1}^n$ in $\mathcal{B}(\mathcal{H}_{\rm A})$ (with $d_{\rm A} = d_{\rm C}$) define the following tripartite separable matrix:

$$\Omega^{ABC} \equiv \sum_{i=1}^n q_i\, \omega_i^{\rm A} \otimes \rho_i^{\rm B} \otimes \sigma_i^{\rm C}\ , \tag{65}$$

and its twirled version

$$\widetilde{\Omega}^{ABC} = \int_G dg \sum_{i=1}^n q_i\, \overline{\mathcal{U}}_g(\omega_i^{\rm A}) \otimes \mathcal{U}_g(\rho_i^{\rm B}) \otimes \mathcal{U}_g(\sigma_i^{\rm C})\ . \tag{66}$$

Then, the following are equivalent:

1. There exists a covariant CPTP map $\mathcal{E} : \mathcal{B}(\mathcal{H}_{\rm B}) \to \mathcal{B}(\mathcal{H}_{\rm C})$ such that
$$\mathcal{E}(\rho_i^{\rm B}) = \sigma_i^{\rm C} \quad \forall\, i = 1, ..., n\ . \tag{67}$$
2. For any $\omega_1^{\rm A}, ..., \omega_n^{\rm A} \in \mathcal{B}(\mathcal{H}_{\rm A})$:
$$2^{-H_{\min}(A|B)_{\widetilde{\Omega}}} \geqslant d_{\rm C}\langle\phi_+^{\rm AC}|\widetilde{\Omega}^{\rm AC}|\phi_+^{\rm AC}\rangle\ . \tag{68}$$
3. For any $\omega_1^{\rm A}, ..., \omega_n^{\rm A} \in \mathcal{B}(\mathcal{H}_{\rm A})$:
$$H_{\min}(A|B)_{\widetilde{\Omega}} \leqslant H_{\min}(A|C)_{\widetilde{\Omega}}\ . \tag{69}$$

Proof. The proof of Lemma 1 goes through unchanged, with the only difference being that we want to find a CPTP map $\mathcal{E}$ that is covariant, i.e., that satisfies the following property:

$$\mathcal{U}_g^{\rm C}[\mathcal{E}(\rho^{\rm B})] = \mathcal{E}(\mathcal{U}_g^{\rm B}[\rho^{\rm B}]) \quad \forall g \in G\ . \tag{70}$$

Hence, we can start from Supplementary Eq. (22), which in the covariant case becomes

$$\begin{aligned}&\forall \text{ states } \{\omega_i^{\rm A}\},\\ &\max_{\mathcal{E}:\text{ covar. CPTP}} \sum_i q_i \operatorname{Tr}\left[\left\{\omega_i^{\rm A} \otimes \mathcal{E}(\rho_i^{\rm B})\right\}\ \phi_+^{\rm AC}\right]\\ &\geqslant \sum_i q_i \operatorname{Tr}\left[\left\{\omega_i^{\rm A} \otimes \sigma_i^{\rm C}\right\}\ \phi_+^{\rm AC}\right]\ .\end{aligned} \tag{71}$$

Using the covariance of the channel Supplementary Eq. (70), and the so-called "ricochet property" of the maximally entangled state, that is, $(\mathbb{1}^{\rm A} \otimes X_{\rm C})|\phi_+^{\rm AC}\rangle = (X_{\rm A}^{\rm T} \otimes \mathbb{1}^{\rm C})|\phi_+^{\rm AC}\rangle)$, we can rewrite the left-hand side of the above inequality as follows:

$$\sum_i q_i \operatorname{Tr}\left[\left\{\omega_i^{\rm A} \otimes \mathcal{E}(\rho_i^{\rm B})\right\}\ \phi_+^{\rm AC}\right]$$
$$= \sum_i q_i \int_G dg \operatorname{Tr}\left[(\omega_i^{\rm A} \otimes \mathcal{E}(\rho_i^{\rm B}))\ (\mathcal{U}_g^{\rm T} \otimes \mathcal{U}_g^\dagger)(\phi_+^{\rm AC})\right] \tag{72}$$
$$= \sum_i q_i \int_G dg \operatorname{Tr}\left[(\overline{\mathcal{U}}_g \otimes \mathcal{U}_g)(\omega_i^{\rm A} \otimes \mathcal{E}(\rho_i^{\rm B}))\ \phi_+^{\rm AC}\right] \tag{73}$$
$$= \langle\phi_+^{\rm AC}|\ (\mathsf{id}_{\rm A} \otimes \mathcal{E}_{\rm B})(\widetilde{\Omega}^{\rm AB})\ |\phi_+^{\rm AC}\rangle\ , \tag{74}$$

where, we recall, the channel $\mathcal{E}$ is assumed to be covariant.

Le us now consider the quantity

$$\max_{\mathcal{E}:\text{CPTP}} \langle\phi_+^{\rm AC}|(\mathsf{id}_{\rm A} \otimes \mathcal{E}_{\rm B})(\widetilde{\Omega}^{\rm AB})|\phi_+^{\rm AC}\rangle\ , \tag{75}$$

where the maximization now is allowed to run over all possible CPTP maps, not only covariant ones. However,

since both $\widetilde{\Omega}^{\rm AB}$ and $\phi_+^{\rm AC}$ are invariant for the action $\overline{\mathcal{U}}_g\otimes\mathcal{U}_g$, we immediately have that

$$\max_{\mathcal{E}:\text{CPTP}}\langle\phi_+^{\rm AC}|(\mathsf{id}_{\rm A}\otimes\mathcal{E}_{\rm B})(\widetilde{\Omega}^{\rm AB})|\phi_+^{\rm AC}\rangle$$

$$=\max_{\mathcal{E}:\text{CPTP}}\operatorname{Tr}\Big[(\mathsf{id}_{\rm A}\otimes\mathcal{E}_{\rm B})(\widetilde{\Omega}^{\rm AB})\ \phi_+^{\rm AC}\Big] \tag{76}$$

$$=\int_G dg\operatorname{Tr}\Big[(\mathsf{id}_{\rm A}\otimes\mathcal{E}_{\rm B})\circ(\overline{\mathcal{U}}_g\otimes\mathcal{U}_g)(\widetilde{\Omega}^{\rm AB})\ (\overline{\mathcal{U}}_g\otimes\mathcal{U}_g)(\phi_+^{\rm AC})\Big] \tag{77}$$

$$=\int_G dg\operatorname{Tr}\Big[(\mathcal{U}_g^{\rm T}\otimes\mathcal{U}_g^\dagger)\circ(\mathsf{id}_{\rm A}\otimes\mathcal{E}_{\rm B})\circ(\overline{\mathcal{U}}_g\otimes\mathcal{U}_g)(\widetilde{\Omega}^{\rm AB})\ \phi_+^{\rm AC}\Big] \tag{78}$$

$$=\int_G dg\operatorname{Tr}\Big[(\mathsf{id}_{\rm A}\otimes\mathcal{U}_g^\dagger\circ\mathcal{E}_{\rm B}\circ\mathcal{U}_g)(\widetilde{\Omega}^{\rm AB})\ \phi_+^{\rm AC}\Big] \tag{79}$$

$$=\max_{\mathcal{E}:\text{ covar. CPTP}}\langle\phi_+^{\rm AC}|(\mathsf{id}_{\rm A}\otimes\mathcal{E}_{\rm B})(\widetilde{\Omega}^{\rm AB})|\phi_+^{\rm AC}\rangle\ , \tag{80}$$

and hence, using the conditional min-entropy,

$$\max_{\mathcal{E}:\text{ covar. CPTP}}\langle\phi_+^{\rm AC}|(\mathsf{id}_{\rm A}\otimes\mathcal{E}_{\rm B})(\widetilde{\Omega}^{\rm AB})|\phi_+^{\rm AC}\rangle \tag{81}$$

$$=\frac{1}{d_{\rm A}}2^{-H_{\min}(A|B)_{\widetilde{\Omega}}}\ . \tag{82}$$

Hence, statement (1) is equivalent to

$$2^{-H_{\min}(A|B)_{\widetilde{\Omega}}} \tag{83}$$

$$\geqslant d_{\rm A}\sum_i q_i\operatorname{Tr}\big[\{\omega_i^{\rm A}\otimes\sigma_i^{\rm C}\}\ \phi_+^{\rm AC}\big] \tag{84}$$

$$=d_{\rm A}\sum_i q_i\operatorname{Tr}\big[\mathcal{G}\{\omega_i^{\rm A}\otimes\sigma_i^{\rm C}\}\ \phi_+^{\rm AC}\big] \tag{85}$$

$$=d_{\rm A}\langle\phi_+^{\rm AC}|\widetilde{\Omega}^{\rm AC}|\phi_+^{\rm AC}\rangle\ . \tag{86}$$

(Remember that $d_{\rm A}=d_{\rm C}$.) Following the same arguments used in the proof of Lemma 1, we also obtain the equivalence between statement (1) and statement (3). This completes the proof of lemma 3.

Remark 2. The existence of a covariant CPTP map achieving the transformation $\rho_i\to\sigma_i$ is of course a stronger requirement than the existence of a general CPTP map doing the same. Indeed, once we rewrite Supplementary Eq. (9) of Lemma 1 as

$$\begin{aligned}2^{-H_{\min}(A|B)_\Omega}&\geqslant d_{\rm C}\langle\phi_+^{\rm AC}|\Omega^{\rm AC}|\phi_+^{\rm AC}\rangle\\&=d_{\rm C}\langle\phi_+^{\rm AC}|\widetilde{\Omega}^{\rm AC}|\phi_+^{\rm AC}\rangle\ ,\end{aligned}$$

and since, as a consequence of the data-processing inequality,

$$2^{-H_{\min}(A|B)_\Omega}\geqslant 2^{-H_{\min}(A|B)_{\widetilde{\Omega}}}\ ,$$

it is clear that it is in principle *harder* to satisfy condition (2) of the covariant Lemma 3, than its non-covariant counterpart (9).

G-covariant version of Theorem 1

As before we used Lemma 1 to prove Theorem 1, here we use Lemma 3 to prove Theorem 2.

Theorem 2 Let $\rho^{\rm AB}\in\mathcal{B}(\mathcal{H}_{\rm A}\otimes\mathcal{H}_{\rm B})$ and $\sigma^{\rm AC}\in\mathcal{B}(\mathcal{H}_{\rm A}\otimes\mathcal{H}_{\rm C})$ be two compatible bipartite quantum states. Denote the dimension of any system X as $d_X\in\mathbb{N}$. The following are equivalent:

1. There exists a G-covariant CPTP map $\mathcal{E}:\mathcal{B}(\mathcal{H}_{\rm A})\to\mathcal{B}(\mathcal{H}_{\rm B})$ such that
$$\sigma^{\rm AC}=\mathsf{id}\otimes\mathcal{E}\left(\rho^{\rm AB}\right) \tag{87}$$

2. For any quantum process (CPTP linear map) $\Phi:\mathcal{B}(\mathcal{H}_{\rm A})\to\mathcal{B}(\mathcal{H}_{\rm A'})$, with $d_{\rm A'}=d_{\rm C}$,
$$\begin{aligned}&H_{\min}(A'|B)_{\mathcal{G}[\Phi\otimes\mathsf{id}(\rho^{\rm AB})]} \qquad (88)\\&\leqslant H_{\min}(A'|C)_{\mathcal{G}[\Phi\otimes\mathsf{id}(\sigma^{\rm AC})]}\ .\end{aligned}$$

3. Supplementary Eq. (88) holds for any measurement-prepare quantum channel $\Phi:\mathcal{B}(\mathcal{H}_{\rm A})\to\mathcal{B}(\mathcal{H}_{\rm A'})$ of the form:
$$\Phi\left(\gamma^{\rm A}\right)=\sum_{j=1}^{d_{\rm A}^2}\operatorname{Tr}\left[M_j^{\rm A}\gamma^{\rm A}\right]\omega_j^{\rm A'}\ , \tag{89}$$
where $\{M_j^{\rm A}\}$ is an arbitrary, but fixed, informationally complete POVM on system A, while the states $\{\omega_j^{\rm A'}\}$ can freely vary.

4. For any $\Phi:\mathcal{B}(\mathcal{H}_{\rm A})\to\mathcal{B}(\mathcal{H}_{\rm A'})$ of the form (89) the following holds:
$$2^{-H_{\min}(A|B)_{\mathcal{G}[\Phi\otimes\mathsf{id}(\rho^{\rm AB})]}} \tag{90}$$
$$\geqslant d_{\rm A}\ \langle\phi^+|\mathcal{G}\left[\Phi\otimes\mathsf{id}\left(\sigma^{\rm AC}\right)\right]|\phi^+\rangle\ , \tag{91}$$
where $|\phi_+^{A'C}\rangle$ is the maximally entangled state between systems A' and C.

We are now ready to prove the main theorem.

Proof of Theorem 2: Let $\{Q_k^{\rm A}\}_{k=1}^{d_{\rm A}^2}$ be the dual basis of $\{M_j^{\rm A}\}$ in $\mathcal{B}(\mathcal{H}_{\rm A})$, that is, $\operatorname{Tr}\left[M_j^{\rm A}Q_k^{\rm A}\right]=\delta_{jk}$. Then, since $\{Q_k^{\rm A}\}$ is itself a basis, we can write

$$\rho^{\rm AB}=\sum_{k=1}^{d_{\rm A}^2}Q_k^{\rm A}\otimes\widetilde{\rho}_k^{\rm B}\quad\text{and}\quad\sigma^{\rm AC}=\sum_{k=1}^{d_{\rm A}^2}Q_k^{\rm A}\otimes\widetilde{\sigma}_k^{\rm C} \tag{92}$$

where

$$\begin{aligned}\widetilde{\rho}_j^{\rm B}&\equiv\operatorname{Tr}_{\rm A}\left[\left(M_j^{\rm A}\otimes\mathbb{1}^{\rm B}\right)\rho^{\rm AB}\right]\\\widetilde{\sigma}_j^{\rm C}&\equiv\operatorname{Tr}_{\rm A}\left[\left(M_j^{\rm A}\otimes\mathbb{1}^{\rm C}\right)\sigma^{\rm AC}\right]\end{aligned} \tag{93}$$

are sub-normalized quantum states (i.e. positive semi-definite matrices). Moreover, since $\rho^{\rm A}=\sigma^{\rm A}$ we have

$\text{Tr}\left[\widetilde{\rho}_j^{\text{B}}\right] = \text{Tr}\left[\widetilde{\sigma}_j^{\text{C}}\right] \equiv p_j$. We therefore conclude that that there exists a covariant CPTP map $\mathcal{E}$ that satisfies (87) if and only if there exists a covariant CPTP map $\mathcal{E}$ that satisfies

$$\sigma_j^{\text{C}} = \mathcal{E}\left(\rho_j^{\text{B}}\right) \tag{94}$$

where $\rho_j^{\text{B}} \equiv \widetilde{\rho}_j^{\text{B}}/p_j$ and $\sigma_j^{\text{C}} \equiv \widetilde{\sigma}_j^{\text{C}}/p_j$. To apply Lemma 3, we introduce a system A' with $d_{\text{A}'} = d_{\text{C}}$, we fix an arbitrary probability distribution $q_i > 0$, and define

$$\Omega^{A'BC} \equiv \sum_{j=1}^{d_{\text{A}}^2} q_j \; \omega_j^{\text{A}'} \otimes \rho_j^{\text{B}} \otimes \sigma_j^{\text{C}} = \sum_{j=1}^{d_{\text{A}}^2} \frac{q_j}{p_j^2} \times \\ \omega_j^{\text{A}'} \otimes \text{Tr}_{\text{A}}\left[\left(M_j^{\text{A}} \otimes I^{\text{B}}\right)\rho^{\text{AB}}\right] \otimes \text{Tr}_{\text{A}}\left[\left(M_j^{\text{A}} \otimes I^{\text{C}}\right)\sigma^{\text{AC}}\right] \, , \tag{95}$$

where the states $\omega_i^{\text{A}'}$ can vary. The corresponding twirled state is

$$\widetilde{\Omega}^{A'BC} \equiv \int_G dg(\overline{\mathcal{U}}_g^{\text{A}} \otimes \mathcal{U}_g^{\text{B}} \otimes \mathcal{U}_g^{\text{C}})(\Omega^{ABC}) \, .$$

Then, taking $q_j = p_j$, we conclude that

$$\widetilde{\Omega}^{A'B} = \mathcal{G}[\Phi \otimes \mathsf{id}\left(\rho^{\text{AB}}\right)] \, , \\ \widetilde{\Omega}^{A'C} = \mathcal{G}[\Phi \otimes \mathsf{id}\left(\sigma^{\text{AC}}\right)] \, . \tag{96}$$

Notice that, in case some $p_i = 0$, we can redefine the measurement operators $M_j^{\text{A}} \to M_j^{\text{A}} + \delta \mathbb{1}^{\text{A}}$, in such a way that they still span the set $\mathcal{B}(\mathcal{H}_{\text{A}})$ but have non-zero probability everywhere. With Supplementary Eq. (96) at hand, the proof of Theorem 2 follows now from Lemma 3.

Covariant Stinespring dilations

Given systems A and A', with Hilbert spaces $\mathcal{H}_{\text{A}}$ and $\mathcal{H}_{\text{A}'}$, we assume that each carry a unitary representation of a compact group G given by $U : G \to \mathcal{B}(H_{\text{A}})$ and $U' : G \to \mathcal{B}(\mathcal{H}_{\text{A}'})$ respectively. A quantum process $\mathcal{E} : \mathcal{B}(A_{\text{A}}) \to \mathcal{B}(\mathcal{H}_{\text{A}'})$ from A into A' is said to be *covariant* or *symmetric* if $\mathcal{E} \circ \mathcal{U}_g = \mathcal{U}'_g \circ \mathcal{E}$ for all $g \in G$. The following lemma was proved in [28], and we provide the proof here for convenience.

Lemma 4 [28] Given a covariant quantum process $\mathcal{E} : \mathcal{B}(\mathcal{H}_{\text{A}}) \to \mathcal{B}(\mathcal{H}_{\text{A}'})$ there exists a Kraus decomposition

$$\mathcal{E}(\rho^{\text{A}}) = \sum_{\lambda,m,k} K_{\lambda,m,k} \rho^{\text{A}} K^{\dagger}_{\lambda,m,k}, \tag{97}$$

with Kraus operators $K_{\lambda,m,k} : \mathcal{H}_{\text{A}} \to \mathcal{H}_{\text{A}'}$ that transform as

$$U^{\text{A}'}(g) K_{\lambda,m,k} U^{\text{A}}(g)^{\dagger} = \sum_j v^{\lambda}(g)_{jk} K_{\lambda,m,j} \tag{98}$$

where $(v^{\lambda}(g)_{jk})$ are the matrix elements of the λ-irrep of G and m is a multiplicity label.

Proof Let $\{K_i\}$ be a set of linearly independent Kraus operators for $\mathcal{E}$. Since $\mathcal{E}$ is covariant we have that $\mathcal{U}_g^{\text{A}'} \circ \mathcal{E} \circ (\mathcal{U}_g^{\text{A}})^{\dagger} = \mathcal{E}$ for any $g \in G$, and so it follows that $\{U^{\text{A}'}(g) K_i U^{\text{A}}(g)^{\dagger}\}_i$ forms another set of Kraus operators for $\mathcal{E}$ for any fixed $g \in G$. Since the Kraus representation is unique up to unitary mixing this implies that $U^{\text{A}'}(g) K_i U^{\text{A}}(g)^{\dagger} = \sum_j V(g)_{ij} K_j$. Moreover, since the Kraus operators are linearly independent it follows that this unitary $V(g)$ is unique for any fixed g and so the matrices $V(g)$ form a non-projective unitary representation of G. Using the unitary freedom to choose the basis $\{K_i\}$ we can choose a basis for which $V(g)$ is block diagonal in terms of a sum of unitary irreps of G. We denote this basis $\{K_{\lambda,m,k}\}$, with $\{K_{\lambda,m,k}\}$ transforming as a λ irrep under G for each multiplicity m as in Equation (98), and k labels the basis vector of the irrep. This completes the proof.

Such Kraus operators are said to transform irreducibly under the group action, and are irreducible tensor operators.

Theorem 3 [Covariant Stinespring [29]] For any covariant quantum process $\mathcal{E} : \mathcal{B}(\mathcal{H}_{\text{A}}) \to \mathcal{B}(\mathcal{H}_{\text{A}'})$ there exists a Stinespring dilation

$$\mathcal{E}(\rho^{\text{A}}) = \text{Tr}_{\text{C}} V(\rho^{\text{A}} \otimes |\sigma\rangle\langle\sigma|^{\text{B}}) V^{\dagger} \tag{99}$$

where $|\sigma\rangle^{\text{B}} \in \mathcal{H}_{\text{B}}$ is a symmetric state under the unitary representation U^{B} of G on system B, system C carries a unitary representation U^{C} of G, and $V : \mathcal{H}_{\text{A}} \otimes \mathcal{H}_{\text{B}} \to \mathcal{H}_{\text{A}'} \otimes \mathcal{H}_{\text{C}}$ is an isometry such that

$$V(U^{\text{A}}(g) \otimes U^{\text{B}}(g)) = (U^{\text{A}'}(g) \otimes U^{\text{C}}(g))V, \tag{100}$$

for all $g \in G$.

Remark 3. This theorem was proved in [29] for the case $\mathcal{H}^{\text{A}} = \mathcal{H}^{\text{A}'}$. The proof of the general case is essentially identical, and we provide the proof below for convenience.

Proof. From the previous lemma, a covariant quantum process $\mathcal{E} : \mathcal{B}(\mathcal{H}_{\text{A}}) \to \mathcal{B}(\mathcal{H}_{\text{A}'})$ always has a Kraus decomposition $\{K_{\lambda,m,k}\}$ such that

$$U^{\text{A}'}(g) K_{\lambda,m,k} (U^{\text{A}})^{\dagger}(g) = \sum_j v^{\lambda}(g)_{jk} K_{\lambda,m,j}, \tag{101}$$

where λ labels an irrep of G, m is a multiplicity label and k is the basis vector label of the irrep.

Let B be a system with Hilbert space $\mathcal{H}_{\text{B}} = \text{span}\{|\sigma\rangle\}$, with the state $|\sigma\rangle$ being symmetric under the action of G. For any pair (λ, m) appearing in the Kraus decomposition of $\mathcal{E}$, let $\mathcal{W}_{(\lambda^*,m)}$ be a Hilbert space isomorphic to the λ^*-irrep of G and for which we choose a basis $\{|\lambda^*, m, k\rangle\}_k$. We define $\mathcal{H}_{\text{C}} := \mathcal{H}_{\text{B}} \bigoplus_{(\lambda,m)} \mathcal{W}_{(\lambda^*,m)}$, where the direct sum ranges over all (λ, m) occurring in the Kraus decomposition of $\mathcal{E}$. The space $\mathcal{H}_{\text{C}}$ carries the unitary group action

$$U^{\text{C}}(g) = |\sigma\rangle\langle\sigma| \bigoplus_{(\lambda,m)} \sum_{j,k} (v^{\lambda}(g)_{jk})^* |\lambda^*, m, j\rangle\langle\lambda^*, m, k|, \tag{102}$$

where $(v^\lambda(g)_{jk})$ are the unitary matrix components of the irrep λ of G.

We define the operator $V : \mathcal{H}_\mathrm{A} \otimes \mathcal{H}_\mathrm{B} \to \mathcal{H}_{\mathrm{A}'} \otimes \mathcal{H}_\mathrm{C}$ as

$$V := \sum_{\lambda,m,k} K_{\lambda,m,k} \otimes |\lambda^*, m, k\rangle\langle\sigma|. \tag{103}$$

Using that the $\{K_{\lambda,m,k}\}_k$ transform irreducibly under the action of G, together with the fact that $(v^\lambda(g)_{jk})$ is a unitary matrix, it is readily verified that Equation (100) holds for all $g \in G$, and so V is covariant under the action of G. Moreover since $\sum_{\lambda,m,k} K^\dagger_{\lambda,m,k} K_{\lambda,m,k} = \mathbb{1}^\mathrm{A}$, and $\{|\lambda^*, m, k\rangle\}_{\lambda,m,k}$ is an orthonormal set of states we have that $V^\dagger V = \mathbb{1}^\mathrm{A} \otimes |\sigma\rangle\langle\sigma|^\mathrm{B}$ and so V is an isometry from $\mathcal{H}_\mathrm{A} \otimes \mathcal{H}_\mathrm{B}$ into $\mathcal{H}_{\mathrm{A}'} \otimes \mathcal{H}_\mathrm{C}$. Finally, we have that

$$\mathcal{E}(\rho^\mathrm{A}) = \mathrm{Tr}_\mathrm{C} V(\rho^\mathrm{A} \otimes |\sigma\rangle\langle\sigma|^\mathrm{B}) V^\dagger, \tag{104}$$

and so have constructed the required dilation for the covariant quantum process $\mathcal{E}$. This completes the proof of theorem.

The following lemma clarifies that any mixed symmetric state can always be purified to a pure quantum state that is also symmetric under the group action.

Lemma 5 Consider a quantum system A, carrying a unitary representation $U^\mathrm{A} : G \to \mathcal{B}(\mathcal{H}^\mathrm{A})$, and a mixed quantum state σ^A for which $\mathcal{U}^\mathrm{A}_g(\sigma^\mathrm{A}) = \sigma^\mathrm{A}$ for all $g \in G$. Then, there exists a purification $|\psi^{\mathrm{AB}}\rangle$ of σ^A onto a composite system AB, and a unitary representation $V^\mathrm{B} : G \to \mathcal{B}(\mathcal{H}^\mathrm{B})$ such that $U^\mathrm{A}_g \otimes V^\mathrm{B}_g |\psi^{\mathrm{AB}}\rangle = |\psi^{\mathrm{AB}}\rangle$ for all $g \in G$.

Since any density operator on $\mathcal{H}^\mathrm{A}$ can be thought as a quantum process from a 1 dimensional input Hilbert space to $\mathcal{B}(\mathcal{H}^\mathrm{A})$, this lemma follows immediately from theorem 3 on Covariant Steinespring dilation. Here, we present a more direct proof.

Let $\{|j\rangle^\mathrm{A}\}^\mathrm{R}_{j=1}$ be an orthonormal basis of the support subspace of σ^A, where r is the rank of σ^A. Let

$$|\psi\rangle^{\mathrm{AB}} = (\sigma^{1/2} \otimes I^\mathrm{B})|\phi_+\rangle^{\mathrm{AB}} \quad ; \quad |\phi_+\rangle^{\mathrm{AB}} \equiv \sum_{j=1}^{\mathrm{R}} |j\rangle^\mathrm{A} |j\rangle^\mathrm{B} \tag{105}$$

be a purification of σ^A. Then,

$$\begin{aligned}
&[U^\mathrm{A}_g \otimes \overline{U}^\mathrm{B}_g]|\psi\rangle^{\mathrm{AB}} \\
=&[U^\mathrm{A}_g \otimes \overline{U}^\mathrm{B}_g][\sqrt{\sigma^\mathrm{A}} \otimes \mathsf{id}_\mathrm{B}]|\phi_+\rangle^{\mathrm{AB}} \\
=&[U^\mathrm{A}_g \otimes \mathsf{id}^\mathrm{B}][\sqrt{\sigma^\mathrm{A}} \otimes \mathsf{id}_\mathrm{B}][\mathsf{id}^\mathrm{A} \otimes \overline{U}^\mathrm{B}_g]|\phi_+\rangle^{\mathrm{AB}} \\
=&[U^\mathrm{A}_g \otimes \mathsf{id}^\mathrm{B}][\sqrt{\sigma^\mathrm{A}} \otimes \mathsf{id}_\mathrm{B}][\overline{U}^{\mathrm{A}^\mathrm{T}}_g \otimes \mathsf{id}^\mathrm{B}]|\phi_+\rangle^{\mathrm{AB}} \\
=&[U^\mathrm{A}_g \sqrt{\sigma^\mathrm{A}} U^{\mathrm{A}^\dagger}_g \otimes \mathsf{id}^\mathrm{B}]|\phi_+\rangle^{\mathrm{AB}} \\
=&[\sqrt{U^\mathrm{A}_g \sigma^\mathrm{A} U^{\mathrm{A}^\dagger}_g} \otimes \mathsf{id}^\mathrm{B}]|\phi_+\rangle^{\mathrm{AB}} \\
=&[\sqrt{\sigma^\mathrm{A}} \otimes \mathsf{id}^\mathrm{B}]|\phi_+\rangle^{\mathrm{AB}} \\
=&|\psi\rangle^{\mathrm{AB}}.
\end{aligned} \tag{106}$$

Therefore, this completes the proof by taking $V_g \equiv \overline{U}^\mathrm{B}_g$.

SUPPLEMENTARY NOTE 6: GENERALIZED THERMAL PROCESSES

We prove the general result in the presence of thermodynamic observables $\{H^\mathrm{A}, X^\mathrm{A}_1, \ldots, X^\mathrm{A}_n\}$, which may have non-trivial commutation relations between them. The case on the Hamiltonian being the only thermodynamic observable follows as a special case of this result.

Assumptions (A1) and (A2), together with the requirement that the resource theory be non-trivial in these observables implies that the free state must take the form of the generalized Gibbs ensemble γ^A,

$$\gamma^\mathrm{A} = \frac{1}{\mathcal{Z}} e^{-\beta(H^\mathrm{A} - \sum_k \mu_k X^\mathrm{A}_k)} \tag{107}$$

for constants $\beta, \mu_1, \ldots, \mu_n$. This is picked out in several different ways, for example perhaps the simplest to interpret is within the theory of equilibration. An alternative route is through a complete passivity argument in which one has additional access to an ordered macroscopic ‘bath’ for each observable that can give or take arbitrary amounts of that observable. Given an unbounded number of copies of the free state one wishes to know if one can trivialise the theory in terms of providing an arbitrary displacement for any of these observables. However in the presence of thermodynamic constraints, these are coupled in such a way that one must only consider an “effective” energy bath with Hamiltonian $\tilde{H} = H - \sum_k \mu_k X_k$. Complete passivity with respect to this observable implies the above generalized Gibbs state through standard arguments.

We now give a precise statement of assumption (A3) in the context of thermodynamic observables $\{H^\mathrm{S}, X^\mathrm{S}_1, \ldots, X^\mathrm{S}_n\}$ for any quantum system S. We first note there are two components to any TP process $\mathcal{E}$ at the microscopic level: the particular interactions between A and an auxiliary system B, and the state σ^B of the auxiliary system. Under assumption (A1) there are no couplings present between eigenspaces of different eigenvalues of the additively conserved observables, however this does not mean that coherence cannot be injected into A. Assumption (A3) places a minimal constraint on the use of coherence sources outside of A. The key idea is that while $\mathcal{E}$ may be realised through some specific interaction V between A and its environment B, and this environment may even contain quantum coherences in its state σ^B, we can guarantee that $\mathcal{E}$ is not exploiting any of these coherences if it is the case that if we were to *remove* the coherences present in σ^B then the transformation $\mathcal{E}$ would still be possible through interactions with B. This motivates the following condition.

(A3) (Incoherence) Given a thermodynamically free process $\mathcal{E} : \mathcal{B}(\mathcal{H}_\mathrm{A}) \to \mathcal{B}(\mathcal{H}_{\mathrm{A}'})$ there exists an interaction isometry W that obeys (133) and a quantum state η^B such that

$$\mathcal{E}(\rho^\mathrm{A}) = \mathrm{Tr}_\mathrm{C} W(\rho^\mathrm{A} \otimes \eta^\mathrm{B}) W^\dagger, \tag{108}$$

and with $\eta^{\rm B} = \mathcal{G}(\eta^{\rm B})$ where

$$\mathcal{G}(\eta^{\rm B}) := \int dg U_{\rm B}(g)\eta^{\rm B} U_{\rm B}(g)^\dagger, \tag{109}$$

and where $U_{\rm B}(g)$ is the group representation on B generated by the observables $\{H^{\rm B}, X_1^{\rm B}, \dots, X_n^{\rm B}\}$. Below in Lemma 7 we show that this assumption captures the demand that no coherence is being exploited from the environment, but before this we establish that the set TP has a compact formulation in terms of covariance.

Lemma 6 Given a set of thermodynamic observables $\{H^{\rm S}, X_1^{\rm S}, \dots, X_n^{\rm S}\}$ for any quantum system S, the set TP of quantum processes from A into A' defined by (A1-A3) coincides with the set GPC of Gibbs-preserving processes on A that are covariant under the group G generated by the thermodynamic observables on A and A'.

Proof We first show that $TP \subset GPC$. Assumption (A2) ensures that the image of the Gibbs state $\gamma^{\rm A}$ under TP is the fixed point $\gamma^{\rm A'}$, so it suffices to establish covariance. For any system S we define $X_0^{\rm S} := H^{\rm S}$ so as to make notation compact. Given a process $\mathcal{E} \in TP$, assumption (A1) implies that

$$\mathcal{E}(\rho^{\rm A}) = {\rm Tr}_{\rm C} V(\rho^{\rm A} \otimes \sigma^{\rm B})V^\dagger, \tag{110}$$

for some V that obeys the conservation laws given by Equation (133). In particular, this implies that

$$V \exp\left[i\sum_{k=0}^{n} \theta_k X_k^{\rm AB}\right] = \exp\left[i\sum_{k=0}^{n} \theta_k X_k^{A'C}\right] V \tag{111}$$

$$X_k^{\rm AB} := X_k^{\rm A} \otimes \mathbb{1}^{\rm B} + \mathbb{1}^{\rm A} \otimes X_k^{\rm B} \tag{112}$$

$$X_k^{A'C} := X_k^{\rm A'} \otimes \mathbb{1}^{\rm C} + \mathbb{1}^{\rm A'} \otimes X_k^{\rm C} \tag{113}$$

for all $k = 0, \dots d$ and for all $\theta_k \in \mathbb{R}$. Therefore the observables $\{X_k\}$ generate a representation of a group G, with elements g indexed by $(\theta_0, \dots, \theta_d)$, and $U^{A'C}(g)V = VU^{\rm AB}(g)$ for all $g \in G$. Therefore the process sending any $\chi^{\rm AB} \to V\chi^{\rm AB}V^\dagger$ is G-covariant. As discussed, assumption (A3) says that the above $\sigma^{\rm B}$ can be taken to be symmetric under this group action: $\mathcal{U}_g^{\rm B}(\sigma^{\rm B}) = \sigma^{\rm B}$. Since discarding systems is G-covariant, and also composing of G-covariant processes results in a G-covariant process, we see that $\rho^{\rm A} \to \rho^{\rm A} \otimes \sigma^{\rm B} \to V(\rho^{\rm A} \otimes \sigma^{\rm B})V^\dagger \to {\rm Tr}_{\rm C} V(\rho^{\rm A} \otimes \sigma^{\rm B})V^\dagger = \mathcal{E}(\rho^{\rm A})$ is a G-covariant process for any $\mathcal{E}$ of the form (110). Therefore $TP \subset GPC$.

Conversely, let $\mathcal{E} \in GPC$. Since $\mathcal{E}(\gamma^{\rm A}) = \gamma^{\rm A'}$, assumption (A2) holds automatically. Since $\mathcal{E}$ is G-covariant with respect to the group generated by $\{X_k^{\rm A}\}$ as shown there exists a Stinespring dilation of the process $\mathcal{E}$ of the form

$$\mathcal{E}(\rho^{\rm A}) = {\rm Tr}_{\rm C} V(\rho^{\rm A} \otimes |\psi\rangle\langle\psi|^{\rm B})V^\dagger, \tag{114}$$

where V is a G-invariant isometry and $|\psi\rangle^{\rm B}$ is invariant under the group action on B. The invariance of V implies that assumption (A1) holds, while the symmetry of $|\psi\rangle$ implies that there are no coherences between eigenspaces of the distinguished observables and so (A3) holds. Therefore $\mathcal{E} \in TP$, and so the two sets of processes coincide as claimed. This completes the proof of lemma.

To summarize, the state interconversion under TPs is equivalent to the following requirement:

$$\mathcal{E}(\rho^{\rm A}) = \sigma^{\rm A'} \tag{115}$$

$$\mathcal{E}(\gamma^{\rm A}) = \gamma^{\rm A'}. \tag{116}$$

where $\mathcal{E}$ is required to be a G-covariant process.

We can now show that no coherences are exploited from the environment for any $\mathcal{E}$ in TP.

Lemma 7 Suppose $\mathcal{E}$ is in TP and realised as

$$\mathcal{E}(\rho^{\rm A}) = {\rm Tr}_{\rm C} V(\rho^{\rm A} \otimes \sigma^{\rm A})V^\dagger, \tag{117}$$

by some isometry V obeying (133 and interacting with a system B in a state $\sigma^{\rm B}$. Then (117) also holds with $\sigma^{\rm B}$ replaced by $\mathcal{G}(\sigma^{\rm B})$.

Proof Since $\mathcal{E}$ is a covariant map, we have that $\mathcal{U}_g[\mathcal{E}(\mathcal{U}_{g^{-1}}(\rho^{\rm A})] = \mathcal{E}(\rho^{\rm A})$ for any $g \in G$. Expressing $\mathcal{E}$ in terms of $(V, \sigma^{\rm B})$ and exploiting the fact that $U_{\rm A'}(g) \otimes \mathbb{1}_{\rm C} V = \mathbb{1}_{\rm A'} \otimes U_{\rm C}(g)^\dagger V U_{\rm A}(g) \otimes U_{\rm B}(g)$ we see that

$$\begin{aligned}\mathcal{U}_g({\rm Tr}_{\rm C} V(\mathcal{U}_{g^{-1}}(\rho^{\rm A}) \otimes \sigma^{\rm B})V^\dagger) &= {\rm Tr}_{\rm C} V(\rho^{\rm A} \otimes \mathcal{U}_g(\sigma^{\rm B}))V^\dagger \\ &= \mathcal{E}(\rho^{\rm A}),\end{aligned} \tag{118}$$

for any $g \in G$. Integrating over all g and using linearity we deduce that

$$\mathcal{E}(\rho^{\rm A}) = {\rm Tr}_{\rm C} V(\rho^{\rm A} \otimes \mathcal{G}(\sigma^{\rm B}))V^\dagger \tag{119}$$

as required.

Remark 4. Note that Lemma 5 shows that replacing any auxiliary $\sigma^{\rm B}$ with its dephased version $\mathcal{G}(\sigma^{\rm B})$ as discussed in the main text is consistent with the existence of a Stinespring form in which the auxiliary system is taken to be in a pure symmetric quantum state. Also note that that we implicitly assume that the group G generated by the thermodynamic observables on the input system coincides with the group generated by those on the output system, which is a basic physical requirement.

SDP solution for thermomajorization with coherence

Here we illustrate how to use SDP to solve the decision problem of determining if $\rho \in {\rm B}(\mathcal{H})$ can be converted to $\sigma \in {\rm B}(\mathcal{H})$ by a generalized thermal processes. For simplicity of the exposition, we consider the case of no charges with the same Hamiltonian for the input and output spaces. We want to know if there exists a thermal

process, that is, a CPTP map $\mathcal{E} : \mathrm{B}(\mathcal{H}) \to \mathrm{B}(\mathcal{H})$ that is both Gibbs preserving and symmetric under time translation, such that $\sigma = \mathcal{E}(\rho)$. Denoting the Hamiltonian by H and the Gibbs state by $\gamma = \frac{1}{Z} e^{-\beta H}$, from (45) it follows that there exists such a Gibbs preserving symmetric map $\mathcal{E}$ if and only if $f(\rho, \sigma) \geqslant 1$, where $f(\rho,\sigma)$ is given by the SDP problem

$$\begin{aligned} f(\rho,\sigma) &\equiv \max\ y \\ \text{subject to: } & \mathbf{1.}\ \tau^{\mathrm{AB}} \geqslant 0\ ;\ \tau^{\mathrm{B}} \leqslant I \\ & \mathbf{2.}\ y\sigma^{\mathrm{T}} \leqslant \mathrm{Tr_B}\left[\tau^{\mathrm{AB}}(I\otimes\rho)\right] \\ & \mathbf{3.}\ y\gamma^{\mathrm{T}} \leqslant \mathrm{Tr_B}\left[\tau^{\mathrm{AB}}(I\otimes\gamma)\right] \\ & \mathbf{4.}\ \mathcal{G}(\tau^{AB}) = \tau^{AB}\ . \end{aligned} \tag{120}$$

Here we added the condition $\mathcal{G}(\tau^{AB}) = \tau^{AB}$ to (45) to ensure that $\mathcal{E}$ is symmetric under time translation with respect to the Hamiltonian H. The G-twirling is given as in (64) with g replaced by the time parameter t, and the group element $\mathcal{U}_t(\cdot) = e^{iHt}(\cdot)e^{-iHt}$.

The dual to this problem is very similar to (37) and the only difference is that one has to take the G-twirling on the term $\sum_{i=1}^n X_i \otimes \rho_i$. For the more specific case we consider here, we have:

$$\begin{aligned} f(\rho,\sigma) &= \min \mathrm{Tr}[Z] \\ \text{subject to } & I\otimes Z \geqslant \mathcal{G}\left(X\otimes\rho\right) + Y\otimes\gamma, \\ & \mathrm{Tr}\left(\sigma^{\mathrm{T}}X\right) + \mathrm{Tr}\left(\gamma^{\mathrm{T}}Y\right) = 1\ ;\ X,Y \geqslant 0 \end{aligned}$$

where we replaced $\mathcal{G}\left(Y\otimes\gamma\right)$ with $Y\otimes\gamma$ since γ is symmetric.

As before, the above minimization problem is an SDP. To see it, we follow now the same steps that led to Supplementary Eq. (42). We define the vector space V_1, consisting of all Hermitian matrices $\zeta \in V_1$ of the form:

$$\zeta = (\eta, Z, X, Y) \tag{121}$$

where $\eta \in \mathrm{B}(\mathcal{H}\otimes\mathcal{H})$, and $X, Y, Z \in \mathrm{B}(\mathcal{H})$ are all Hermitian. In addition, we define the vector space V_2 of all Hermitian matrices in $\mathrm{B}(\mathcal{H}\otimes\mathcal{H})$, and a linear transformation $\Gamma : V_1 \to V_2$ given by:

$$\Gamma\left(\zeta\right) = I\otimes Z - \mathcal{G}\left(X\otimes\rho\right) - Y\otimes\gamma - \eta\ . \tag{122}$$

Clearly, the map above is linear. Set $\tau \equiv (\mathbf{0}, \mathbf{0}, \sigma^{\mathrm{T}}, \gamma^{\mathrm{T}})$ so that $\mathrm{Tr}[\sigma\zeta] = \mathrm{Tr}\left(\sigma^{\mathrm{T}}X\right) + \mathrm{Tr}\left(\gamma^{\mathrm{T}}Y\right)$. We also denote $C \equiv (\mathbf{0}, I, \mathbf{0}, \mathbf{0})$. With these notations:

$$f(\rho,\sigma) = \min\left\{\mathrm{Tr}[C\zeta]\ \Big|\ \zeta \geqslant 0\ ;\ \Gamma(\zeta) = 0\ ,\ \mathrm{Tr}[\sigma\zeta] = 1\right\} \tag{123}$$

To bring the above optimization problem to a canonical SDP form, we denote as before $H_j \equiv \Gamma^*(E_j)$, where $j = 1, ..., d^4$, where $d \equiv \dim(\mathcal{H})$, and E_j is a basis of $\mathrm{B}(\mathcal{H}\otimes\mathcal{H})$. We also denote $H_0 \equiv \tau$. With this notations we get

$$\begin{aligned} f(\rho,\sigma) &= \min \mathrm{Tr}[C\zeta] \\ \text{subject to } & \zeta \geqslant 0\ ,\ \mathrm{Tr}\left(\zeta H_j\right) = \delta_{0,j} \quad \forall j \in \{0, 1, ..., d^4\}\ . \end{aligned} \tag{124}$$

The above optimization form is written in a canonical form and can be solved with SDP packages such as CVX.

SUPPLEMENTARY NOTE 7: PROOF OF THEOREMS 2 AND 3

Theorem 2 is a special case of theorem 3, where the only additive conserved observable is the Hamiltonian (See remark 5). Therefore, it suffices to prove theorem 3.

The proof is basically a corollary of lemma 3. For any system $S = R, A, A'$, let $\{U^{\mathrm{S}}(g)\}$ be the symmetry group generated by the additively conserved observables $\{H^{\mathrm{S}}, X_k^{\mathrm{S}};\ k = 1, \ldots, n\}$, where for system R we define

$$H^{\mathrm{R}} = -(H^{\mathrm{A'}})^{\mathrm{T}}, \text{ and } X^{\mathrm{R}} = -(X^{\mathrm{A'}})^{\mathrm{T}}\ , \tag{125}$$

and the superscript T denotes the transpose. Note that this definition implies that for any group element g,

$$U^{\mathrm{R}}(g) = \overline{U}^{\mathrm{A'}}(g)\ . \tag{126}$$

We are interested to determine if there exists a CPTP map $\mathcal{E} :\ \mathcal{B}(\mathcal{H}^{\mathrm{A}}) \to \mathcal{B}(\mathcal{H}^{\mathrm{A'}})$ which (i) satisfies

$$\mathcal{E}(\rho^{\mathrm{A}}) = \sigma^{\mathrm{A'}}, \text{ and } \mathcal{E}(\gamma^{\mathrm{A}}) = \gamma^{\mathrm{A'}}\ , \tag{127}$$

and (ii) is covariant, i.e. for all group elements g,

$$\mathcal{E}\circ\mathcal{U}_g^{\mathrm{A}} = \mathcal{U}_g^{\mathrm{A'}}\circ\mathcal{E}\ ,$$

where $\mathcal{U}_g^{\mathrm{S}}[\cdot] = U^{\mathrm{S}}(g)[\cdot]U^{\dagger}(g)$ for any system $S = A, A', R$.

Therefore, to apply lemma 3, we assume systems A, B and C in the statement of lemma 3 correspond, respectively, to systems R, A and A' in the statement of theorem 3. Furthermore, to apply the lemma we assume the set of input states $\{\rho_i\}$ has two elements, namely $\{\rho^{\mathrm{A}}, \gamma^{\mathrm{A}}\}$, and the corresponding output states are $\{\sigma_i\} = \{\sigma^{\mathrm{A'}}, \gamma^{\mathrm{A'}}\}$. Finally, in the statement of lemma 3 states $\{\omega_i\}$ denote possible states of the reference system. Here, we denote them by $\{\eta_1^{\mathrm{R}}, \eta_2^{\mathrm{R}}\}$. Under these assignments state Ω^{ABC} in the statement of lemma becomes

$$\begin{aligned} &\widetilde{\Omega}^{RAA'} \\ &= \int_G dg\, \mathcal{U}_g^{RAA'}[q\eta_1^{\mathrm{R}}\otimes\rho^{\mathrm{A}}\otimes\sigma^{\mathrm{A'}} + (1-q)\eta_2^{\mathrm{R}}\otimes\gamma^{\mathrm{A}}\otimes\gamma^{\mathrm{A'}}]\ , \end{aligned}$$

Then, the equivalence of statements (1) and (3) in lemma 3 implies that the necessary and sufficient condition for existence of a G-covariant CPTP map which satisfies Supplementary Eq.(127) is

$$H_{\min}(R|A)_{\Omega} \leqslant H_{\min}(R|A')_{\Omega}\ . \tag{128}$$

This completes proof of theorem 3.

Remark 5. Note that in the case of theorem 2, where the only additive conserved observable is the Hamiltonian, the G-twirling operation $\int dg\, \mathcal{U}_g$ reduces to the time average

$$\int dg\, \mathcal{U}_g^{\mathrm{S}}[X^{\mathrm{S}}] = \lim_{T\to\infty} \frac{1}{T} \int_{-\frac{1}{2}T}^{\frac{1}{2}T} dt X^{\mathrm{S}}(t) := \langle X^{\mathrm{S}} \rangle \ , \quad (129)$$

where $X^{\mathrm{S}}(t)$ is the time evolved version of X^{S}, i.e. $X^{\mathrm{S}}(t) = e^{-iH^{\mathrm{S}}t} X^{\mathrm{S}} e^{iH^{\mathrm{S}}t}$ for any system S.

SUPPLEMENTARY NOTE 8: RELATION BETWEEN GENERALIZED THERMAL PROCESSES AND THERMAL OPERATIONS

Thermal operations (TO) are defined as quantum operations that take the form

$$\mathcal{E}(\rho^{\mathrm{A}}) = \mathrm{Tr}_{\mathrm{B}} V(\rho^{\mathrm{A}} \otimes \gamma^{\mathrm{B}}) V^{\dagger} \quad (130)$$

where V commutes with the total Hamiltonian of AB, and $\gamma^{\mathrm{B}} = \exp[-\beta H^{\mathrm{B}}]/Z$ is the Gibbs state on B. In contrast, the situation of thermal processes with just a Hamiltonian have a similar form, but now with γ^{B} replaced with a potentially non-Gibbsian incoherent state σ^{B} and with the condition that $\mathcal{E}(\gamma^{\mathrm{A}}) = \gamma^{\mathrm{A}}$ being imposed independently.

One might think that the two sets are equivalent and that every TP admits a form given by (130), but this turns out to not be the case. For example, if we consider the case of trivial Hamiltonians $H^{\mathrm{A}} = 0$ then TO is the set of noisy operations, whereas TP is the set of *unital* operations. It is well known that these two sets are not the same [18]. That said, the state interconversion conditions for noisy operations and unital operations are in fact the same – namely majorization on the eigenvalues of the states. One might then conjecture that TO and TP have the same "power" in the sense that $\rho \longrightarrow \sigma$ is possible under TO if and only if it is possible under TP. It can be seen that this is in fact the case if either state is incoherent in energy, since the necessary and sufficient conditions are simply thermo-majorization [17]. However for fully coherent states we do not have a clear understanding of how the two classes compare.

It is also clear that TO is a proper subset of TP, since every TO operation obeys (A1-A3), however there is at present no obvious coherent scenario that could be used to say that one of the two classes is preferred over the other.

In the case of multiple conserved quantities beyond the Hamiltonian, a similar comparison can be made. However in this case the formulation of TO faces subtleties since it relies on a notion of a *free state* which can be prepared in an unbounded number of copies, whereas in contrast TP only makes a statement on the existence of an *equilibrium state*. The free states in TO are singled out because they are completely passive and so are the only form of state admissible that does not trivialise the energetic degrees of freedom. However when introduces multiple conserved quantities $\{H^{\mathrm{B}}, X_1^{\mathrm{B}}, X_2^{\mathrm{B}}, \dots\}$ the question of complete passivity becomes more problematic (see [15, 24–27] for discussion). In general it is impossible for a state to be completely passive in all of the individual observables, and to circumvent this one must define an "effective Hamiltonian" and impose complete passivity solely on this. While this does lead to the generalized Gibbs ensemble, the effective Hamiltonian is not determined by the resource theory framework, but must be postulated independently. In contrast, TP does not require any complete passivity analysis for the bath, since it does not specify a set of free states in the sense described, but instead only demands the existence of an equilibrium state γ^{A} on the primary system. In these terms, the generalized Gibbs ensemble has been shown to arise naturally in the context of equilibration theory (see for example [19] and references therein).

SUPPLEMENTARY NOTE 9: FINITE PRECISION AND APPROXIMATE ENERGY INCOHERENCE

We can replace assumption (A3) with a slightly weaker version that takes into account that we only ever experimentally probe to some finite level of precision. The reason this is useful is that it avoids two technicalities: firstly that the time-translation group action is in general non-compact group $\mathbb{R}$, and secondly even if time-translation is the compact $U(1)$ Lie group no finite dimensional representations will exist in which one can encode all group elements into perfectly distinguishable quantum states. We can circumvent both of these technicalities with the following finite precision assumptions.

Firstly, we can always approximate any quantum system with one having finite dimension $d < \infty$, for d sufficiently large. Given this finite dimension, any spectrum $\{E_1, E_2, \dots, E_d\}$ for the system's Hamiltonian H^{A} can be approximated to an arbitrary precision by a set of rational numbers, $\{\tilde{E}_1 = \frac{a_1}{b_1}, \dots, \tilde{E}_d = \frac{a_d}{b_d}\}$ with $a_k, b_k \in \mathbb{Z}$ for each k and $\tilde{E}_k$ arbitrarily close to E_k. Thus, for simplicity we assume the Hamiltonian has a spectrum of rational numbers and so the resultant unitary dynamics $U^{\mathrm{A}}(t) = \exp[-itH^{\mathrm{A}}]$ is periodic for some finite period $\tau < \infty$.

The mapping $t \mapsto U^{\mathrm{A}}(t)$ is therefore a unitary representation of the continuous $U(1)$ group on the system A. We may further assume that we only ever resolve time intervals $[t_1, t_2]$ with $t_2 - t_1 \geqslant \epsilon$ for some small yet finite level of precision $\epsilon > 0$. More formally this means that we can replace the $U(1)$ group with the discrete $\mathbb{Z}_N$

action, where $N\epsilon = \tau$ and

$$n \mapsto U^{\mathrm{A}}(n\epsilon) = e^{-in\epsilon H^{\mathrm{A}}}, \tag{131}$$

with $n = 0, 1, \ldots N-1$. Therefore the dynamics of any single quantum system can always be approximated by such a discrete, finite action for some $N \in \mathbb{N}$ and sufficiently large.

In the case that we have multiple systems $A_1, A_2, \ldots, A_M$ with periods $\tau_1, \tau_2, \ldots, \tau_M$ respectively, we may choose $\tau = \prod_{k=1}^{M} \tau_k$ as the time-scale for the composite system. Therefore for multiple systems, there will always exist an $N \in \mathbb{N}$, sufficiently large so that the mapping $n \mapsto \exp[-in\epsilon H^{A_k}]$ is a unitary representation of $\mathbb{Z}_N$ on each $\mathcal{H}_{A_k}$, and which approximates the unitary dynamics of each A_k under its Hamiltonian to the specified level of precision. Given this, condition (A3) for incoherence of thermal processes can be replaced with the following.

(A3′) Approximate incoherence. Consider the case of the Hamiltonian being the only thermodynamic observable, and assume the finite precision approximations described above. If the thermodynamically free process $\mathcal{E} : \mathcal{B}(\mathcal{H}_{\mathrm{A}}) \to \mathcal{B}(\mathcal{H}_{\mathrm{A}'})$ is realized microscopically as

$$\mathcal{E}(\rho^{\mathrm{A}}) = \mathrm{Tr}_{\mathrm{C}} V(\rho^{\mathrm{A}} \otimes \sigma^{\mathrm{B}})V^{\dagger}, \tag{132}$$

with V obeying Equation

$$\begin{aligned} V(H^{\mathrm{A}} \otimes \mathbb{1}^{\mathrm{B}} + \mathbb{1}^{\mathrm{A}} \otimes H^{\mathrm{B}}) &= (H^{\mathrm{A}'} \otimes \mathbb{1}^{\mathrm{C}} + \mathbb{1}^{\mathrm{A}'} \otimes H^{\mathrm{C}})V \\ V(X_k^{\mathrm{A}} \otimes \mathbb{1}^{\mathrm{B}} + \mathbb{1}^{\mathrm{A}} \otimes X_k^{\mathrm{B}}) &= (X_k^{\mathrm{A}'} \otimes \mathbb{1}^{\mathrm{C}} + \mathbb{1}^{\mathrm{A}'} \otimes X_k^{\mathrm{C}})V \ , \end{aligned} \tag{133}$$

then we also have

$$\mathcal{E}(\rho^{\mathrm{A}}) = \mathrm{Tr}_{\mathrm{C}} W(\rho^{\mathrm{A}} \otimes \mathcal{G}_\epsilon(\sigma^{\mathrm{B}}))W^{\dagger}. \tag{134}$$

with $\mathcal{G}_\epsilon(\sigma^{\mathrm{B}})$ being the group average over $\mathbb{Z}_N$ of the state σ^{B} given by

$$\mathcal{G}_\epsilon(\sigma^{\mathrm{B}}) := \frac{1}{N}\sum_{n=0}^{N-1} U_\epsilon^{\mathrm{B}}(n)\sigma^{\mathrm{B}} U_\epsilon^{\mathrm{B}}(n)^{\dagger}, \tag{135}$$

with $U_\epsilon^{\mathrm{B}}(n) := \exp[-in\epsilon H^{\mathrm{B}}]$ is the finite precision time evolution on B, and we interact this state with A through some potentially different isometry W that also obeys (133).

This implies that the constraint of time-translation covariance is replaced with $\mathbb{Z}_N$-covariance to this level of precision. Given this, the analysis for state interconversion may be repeated under $(A3')$ and results in the replacement of $\frac{1}{\tau}\int_0^\tau dt(\cdot)$ with $\frac{1}{N}\sum_{k=0}^{N-1}(\cdot)$ and $U^{\mathrm{R}}(t) \otimes U^{\mathrm{A}}(t)$ by the discrete approximation $U_\epsilon^{\mathrm{R}}(n) \otimes U_\epsilon^{\mathrm{A}}(n)$.

SUPPLEMENTARY NOTE 10: CLOCK TIMES AND GUESSING PROBABILITIES

As in the previous section, we may restrict our attention to a fully discrete setting with quantum systems of finite dimension and finite level of precision ϵ for time resolution. Covariance of the dynamics is now described with respect to the discrete group $\mathbb{Z}_N$ for some sufficiently large $N \in \mathbb{N}$.

For $q \to 1$ we obtain the $\mathbb{Z}_N$ covariance constraint alone, and the corresponding state Ω^{RA} takes the form

$$\Omega^{\mathrm{RA}} = \frac{1}{N}\sum_{k=0}^{N-1} U_\epsilon^{\mathrm{R}}(n)\eta_1^{\mathrm{R}}(U_\epsilon^{\mathrm{R}}(n))^{\dagger} \otimes U_\epsilon^{\mathrm{A}}(n)\rho^{\mathrm{A}}(U_\epsilon^{\mathrm{A}}(n))^{\dagger}. \tag{136}$$

For a sufficiently large reference frame R there exists a Hamiltonian H^{R} such that R allows a perfect encoding of the group elements of $G = \mathbb{Z}_N$. In particular for $\dim(\mathcal{H}_{\mathrm{R}}) = \mathrm{N}$ with orthonormal basis $\{|E_k\rangle^{\mathrm{R}}\}$, we can choose

$$U_\epsilon^{\mathrm{R}}(1) = \sum_{k=0}^{N-1} \omega^k |E_k\rangle\langle E_k|^{\mathrm{R}}, \tag{137}$$

where $\omega := e^{\frac{2\pi i}{N}}$ is an Nth root of unity. We then have that $(U_\epsilon^{\mathrm{R}}(1))^n = U_\epsilon^{\mathrm{R}}(n)$ for any $n = 1, 2, \ldots$ and $U_\epsilon^{\mathrm{R}}(N) = U_\epsilon^{\mathrm{R}}(0) = \mathbb{1}^{\mathrm{R}}$ as required.

Defining $|k\rangle^{\mathrm{R}} := F|E_k\rangle^{\mathrm{R}}$, with F being the discrete Fourier transform operator

$$F = \frac{1}{\sqrt{N}}\sum_{i,j=0}^{N-1} \omega^{ij}|E_i\rangle\langle E_j|^{\mathrm{R}}, \tag{138}$$

it is readily seen that

$$U_\epsilon^{\mathrm{R}}(n)|0\rangle^{\mathrm{R}} = |n\rangle^{\mathrm{R}}, \tag{139}$$

and $\langle n|m\rangle^{\mathrm{R}} = 0$ for $n \neq m$ and equal to 1 for $n = m$. Therefore the reference system R provides a perfect classical encoding of the group elements of $\mathbb{Z}_N$ in the pure states $\{|k\rangle^{\mathrm{R}}\}$.

Setting $\eta_1^{\mathrm{R}} = |0\rangle\langle 0|^{\mathrm{R}}$ in equation 136 gives the classical-quantum state

$$\Omega^{\mathrm{RA}} = \frac{1}{N}\sum_{k=0}^{N-1} |k\rangle\langle k|^{\mathrm{R}} \otimes \rho^{\mathrm{A}}(n). \tag{140}$$

where we define $\rho^{\mathrm{A}}(n) := U_\epsilon^{\mathrm{A}}(n)\rho^{\mathrm{A}}(U_\epsilon^{\mathrm{A}}(n))^{\dagger}$ for the state of A at time $t = n\epsilon$. These states fully encode the set of *clock times* $t = 0, \epsilon, \ldots, n\epsilon, \ldots, (N-1)\epsilon$ for the joint system.

Since Ω^{RA} is a classical-quantum state, we have that [5]

$$H_{\min}(R|A)_\Omega = -\log p_{\mathrm{guess}}, \tag{141}$$

where p_{guess} is the optimal Helstrom guessing probability for the ensemble of states $\{(\frac{1}{N}, \rho^{\mathrm{A}}(n))\}_{n=0}^{N-1}$ on A. This

implies that $2^{-H_{\min}(R|A)_\Omega}$ is the optimal guessing probability of the clock time $t = n\epsilon$ for the joint system, given the single copy of ρ^{A}. Monotonicity of $H_{\min}(R|A)_\Omega$ under the thermal processes implies monotonicity of the clock time guessing probability for the system.

REFERENCES

[1] R.T. Rockafellar, *Convex Analysis*, Princeton University Press (1970).

[2] F. G. S. L. Brandão, M. Horodecki, N. H. Y. Ng, J. Oppenheim, S. Wehner, *The second laws of quantum thermodynamics*, PNAS, **112**, 3275 (2015).

[3] G. Gour, *Infinite number of conditions for local mixed state manipulations.* Phys. Rev. A **72**, 022323 (2005).

[4] M. Lostaglio, D. Jennings, T. Rudolph, *Description of quantum coherence in thermodynamic processes requires constraints beyond free energy*, Nat. Comm., **6**, 6383 (2015).

[5] R. König, R. Renner, and C. Schaffner, IEEE Transactions on Information Theory, **55** (9), 4337 (2009).

[6] R. Renner, *Security of Quantum Key Distribution*, Ph.D. Thesis, Diss. ETH No. 16242; pre-print at arXiv:quant-ph/0512258 (2005).

[7] M. Tomamichel, *Quantum Information Processing with Finite Resources - Mathematical Foundations*, Springer Briefs in Mathematical Physics (2016).

[8] D. Blackwell, *Comparison of experiments.* In *Proc. 2nd Berkeley Symposium on Mathematical Statistics and Probability*, 93-102 (1951).

[9] S. Sherman, *On a theorem of Hardy, Littlewood, Pólya and Blackwell*, Proc. Nat. Acad. Sciences **37**, 826-831 (1951).

[10] C. Stein, *Notes on a Seminar on Theoretical Statistics. I. Comparison of experiments*, Report, University of Chicago (1951).

[11] D. Blackwell, *Equivalent comparisons of experiments.* Ann. Math. Stat. **24**, 265-272 (1953).

[12] E. Torgersen, *Comparison of experiments when the parameter space is finite*, Z. Wahrscheinlichkeitstheorie verw. Geb. 16, 219-249 (1970).

[13] E. Ruch, R. Schranner, and T.H. Seligman, *Generalization of a Theorem by Hardy, Littlewood, and Pólya*, J. Math. Analysis and Applications, **76**, 222 (1980).

[14] F. Buscemi, *Comparison of Quantum Statistical Models: Equivalent Conditions for Sufficiency*, Commun. Math. Phys. **310**, 625-647 (2012).

[15] M. Lostaglio, D. Jennings, T. Rudolph, *Thermodynamic resource theories, non-commutativity and maximum entropy principles*, New Journal of Physics **19** 043008 (2017).

[16] N. Yunger Halpern, O. Faist, J. Oppenheim, A. Winter, *Microcanonical and resource-theoretic derivations of the thermal state of a quantum system with noncommuting charges*, Nat. Comm., **7**, 12051 (2016).

[17] M. Horodecki, J. Oppenheim, *Fundamental limitations for quantum and nanoscale thermodynamics*, Nat. Commun., volume **4**, 2059 (2013).

[18] G. Gour, M.P. Mueller, V. Narasimhachar, R.W. Spekkens, N. Yunger Halpern, *The resource theory of informational nonequilibrium in thermodynamics*, Phys. Rep. **583**, 1-58 (2015).

[19] A. C. Cassidy, C. W. Clark, M. Rigol, *Generalized Thermalization in an Integrable Lattice System*, Phys. Rev. Lett. **14** 140405, (2011).

[20] G. Dahl, *Matrix majorization*, Linear Algebra and its Applications, **288**, 53 (1999).

[21] F. Buscemi, *Degradable Channels, Less Noisy Channels, and Quantum Statistical Morphisms*, Problems of Information Transmission, **53** (3), 201 (2016).
G. Gour, *Quantum resource theories in the single-shot regime*, Phys. Rev. A **95**, 062314 (2017).

[22] P. M. Alberti and A. Uhlmann. *A problem relating to positive linear maps on matrix algebras*, Reports on Mathematical Physics **18**(2), 163-176 (1980).

[23] K. Matsumoto, *An example of a quantum statistical model which cannot be mapped to a less informative one by any trace preserving positive map*, pre-print at arXiv:1409.5658 (2014).

[24] N. Yunger Halpern, *Beyond heat baths II: Framework for generalized thermodynamic resource theories*, pre-print at arXiv:1409.7845 (2014).

[25] N. Yunger Halpern, J. Renes, *Beyond heat baths: Generalized resource theories for small-scale thermodynamics*, Phys. Rev. E **93** 022126 (2016).

[26] N. Yunger Halpern, O. Faist, J. Oppenheim, A. Winter, *Microcanonical and resource-theoretic derivations of the thermal state of a quantum system with noncommuting charges*, Nat. Comm., **7**, 12051 (2016).

[27] Y. Guryanova, S. Popescu, A. J. Short, R. Silva, P. Skrzypczyk, *Thermodynamics of quantum systems with multiple conserved quantities*, Nature communications **7**, 12049 (2016).

[28] G. Gour, R.W. Spekkens, *The resource theory of quantum reference frames: manipulations and monotones*, New J. Phys., **10**, 033023 (2008).

[29] I. Marvian, *Symmetry, Asymmetry and Quantum Information*, PhD thesis, University of Waterloo, https://uwspace.uwaterloo.ca/handle/10012/7088 (2012).

[30] S. Turgut, *Catalytic Transformations for Bipartite Pure States*, J. Phys. A: Math. Theor. 40,12185-12212 (2007).
